# Supplementary material for: Global satellite survey reveals uncertainty in landfill methane emissions
Source: Nature. 2025 Nov 5;647(8089):397–402. doi: 10.1038/s41586-025-09683-8 (PMC12611785; doi:10.1038/s41586-025-09683-8)
Supplement: Supplementary file 1 — Supplementary Methods 1 and 2, Notes 1–11, Figs. 1–22 and Tables 1–10. [file 41586_2025_9683_MOESM1_ESM.pdf]

nature portfolio

https://doi.org/10.1038/s41586-025-09683-8

Supplementary information

Global satellite survey reveals uncertainty in landfill methane emissions

In the format provided by the authors and unedited

Supplementary Information

Global satellite survey reveals uncertainty in landfill methane emissions

Matthieu Dogniaux[1], Joannes D. Maasakkers[1], Marianne Girard[2], Dylan Jervis[2], Jason McKeever[2], Berend J. Schuit[1,2], Shubham Sharma[1], Ana Lopez-Noreña[1], Daniel J. Varon[3] and Ilse Aben[1]

[1]SRON Space Research Organisation Netherlands, Leiden, Netherlands
[2]GHGSat, Inc., Montreal, Canada
[3]School of Engineering and Applied Sciences, Harvard University, Cambridge MA, USA

Corresponding author email: M.Dogniaux@sron.nl

Table of Contents

Supplementary Methods 1: Surface activity detection in waste disposal sites from Sentinel-2 clear sky image timeseries *2*

Supplementary Methods 2: Comparison of GHGSat-based plume sources and Sentinel-2 detected surface activity *6*

Supplementary Note 1: Discussion of plume-based IME estimates of emission rates against atmospheric inversion results *8*

Supplementary Note 2: Supplementary visuals, information and data on TROPOMI-based total urban-scale methane emissions *9*

Supplementary Note 3: Explanations on TROPOMI urban hotspot coverage *12*

Supplementary Note 4: Supplementary visuals and information on GHGSat-based facility-scale methane emissions *15*

Supplementary Note 5: Analysis of possible meteorological drivers and seasonality of methane emissions from waste disposal sites *22*

Supplementary Note 6: Comparison of managed landfills and dumping site averaged site-wise emission rate distributions *23*

Supplementary Note 7: Bottom-up dataset filters *29*

Supplementary Note 8: Supplementary results for GHGSat comparison to facility-scale reported and calculated emission rates *33*

Supplementary Note 9: Analyzing the impact of reporting method choice for US facilities *36*

Supplementary Note 10: Results of GHGSat plume sources comparison with Sentinel-2 detected surface activity *37*

Supplementary Note 11: example of detected plumes arising from other facilities than landfills *43*

Supplementary Methods 1: Surface activity detection in waste disposal sites from Sentinel-2 clear sky image timeseries

This supplement describes the algorithm developed for surface activity detection in Sentinel-2 RGB imagery data.

Waste disposal site masks

We outline waste disposal site boundaries using the latest available Google Earth imagery and conduct all the following surface activity analysis within the obtained boundaries, hereafter called "site masks". We also use these boundaries to calculate A, the site area.

Data download

We download Sentinel-2 RGB imagery at 10-m resolution from the Google Earth Engine "COPERNICUS/S2_SR_HARMONIZED" collection in 0.04°x0.04° square images centered on each landfill location, and only include images that show:

- CLOUDY_PIXEL_PERCENTAGE lower than 1%
- A percentage of medium and high probability of cloudy pixels (Surface Classification (SCL) types equal to 8 and 9) within the landfill mask of less than 5%

For each site, the images we use are comprised within 60 days before the first GHGSat observation and 60 days after the last GHGSat observation. In total, we could download Sentinel-2 data that pass these criteria for 119 sites of the 151 observed by GHGSat.

Additional water pixel masking

For each timestamp t, we compute the 5th reflectance percentile in the 12th band (B12) of Sentinel-2 (around 2.2 µm) for all the pixels contained in the site mask. If this 5th reflectance percentile is below 0.1, we preliminary mask all pixels with a B12 reflectance below this value as water pixels. We add a 5-pixel buffer around this preliminary mask to obtain the final water mask.

Additional cloudy pixel masking

For each timestamp t, we preliminary mask all pixels contained in the site mask with Scene Classification (SCL) types equal to 3 (cloud shadow), 8 (clouds medium probability), 9 (clouds high probability) and 10 (cirrus) as cloudy pixels. We add a 7-pixel buffer around this preliminary mask to obtain the final cloudy pixel mask.

Activity detection algorithm

The activity detection algorithm includes several steps, as illustrated in Supplementary Figure 1.

1. We first convert each RGB image to Grayscale RGB (GRGB) by using the NTSC formula: GRGB = 0.299xR + 0.587xG + 0.114xB

2. For a given timestamp t (panels a', b', e, f, g and h in Supplementary Figure 1), we yield the two local Structural SIMilarity maps (SSIM) obtained between images at timestamps t-1 and t (c), and images at timestamps t and t+1 (c').

For both local SSIM maps, we select as most dissimilar the pixels for which SSIM are below or equal to a given percentile P of their respective SSIM distributions (d, d'). We just consider pixels within site boundaries, and exclude pixels additionally masked as water in images at timestamps t-1 and t, and t and t+1, respectively. We use as empirically determined percentile thresholds:

- $P = 5$, if the landfill area $A > 1$ km^2
- $P = 7.5$, if the landfill area $A \leq 1$ km^2

3. We obtain surface activity for timestamp t (e) as the intersection of most dissimilar pixels selected from local SSIM maps obtained between timestamps t-1 and t (c, d) and timestamps t and t+1 (c', d')

4. This three-timestamp moving-window does not allow to obtain activity for the first and last images at timestamps t0 and tmax, respectively.

   Surface activity for the first image at timestamp t0 is identified as the dissimilar pixels selected in the local SSIM map between timestamps t0 and t0+1 that are not included in activity detected for timestamp t0+1.

   Surface activity for the last image at timestamp tmax is identified as the dissimilar pixels selected in the local SSIM map between timestamps tmax-1 and tmax that are not included in activity detected for the timestamp tmax-1.

5. For each timestamp t, we mask out pixels associated with water and clouds (see descriptions of additional water and cloud masking, panel f in Supplementary Figure 1).

6. For each timestamp t, we smooth the remaining pixels associated with surface activity with a median filter using neighborhood sizes dependent on the site size:
   - 50x50 m^2, if the site area $A > 1$ km^2
   - 30x30 m^2, if the site area $A \leq 1$ km^2

   We then perform a binary dilation of the smooth binary activity map Nd times, with:
   - Nd = 5, if the site area $A > 1$ km^2
   - Nd = 3, if the site area $A \leq 1$ km^2

   The result of these two operations highlights spatially consistent activity within the site boundaries (panel g in Supplementary Figure 1).

7. We finally identify individual activity clusters and delineate them with convex hulls, which are compared to GHGSat plume origins (panel h in Supplementary Figure 1).

To ensure the surface activity detection quality, we examine results obtained for each site. We evaluate how the automatically detected surface activity matches what can be visually noticed in RGB and GRGB images. We exclude from the analysis sites where the metric poorly captures real surface activity. For example, those can be caused by:

- A small number of available Sentinel-2 images that result in large temporal gaps between images.

- Miss-classifications in the Sentinel-2 Surface Classification (SCL) product, wrongfully identifying pixels as cloudy or non-cloudy.
- Spurious snowy surfaces that do not compare well with non-snowy images.
- No significant surface activity, leading to insignificant features being identified as surface activity (due to the relative SSIM threshold we use).

Over the 119 sites with sufficient Sentinel-2 data, we keep 107 where we can capture surface activity. Some artefacts can remain in these results. For example, these can be associated with different turbid leachate water colors that challenge the additional water masking, or linked to small orthorectification errors that affect elevated features in sites that may not be resolved by the Digital Elevation Model used for orthorectification.

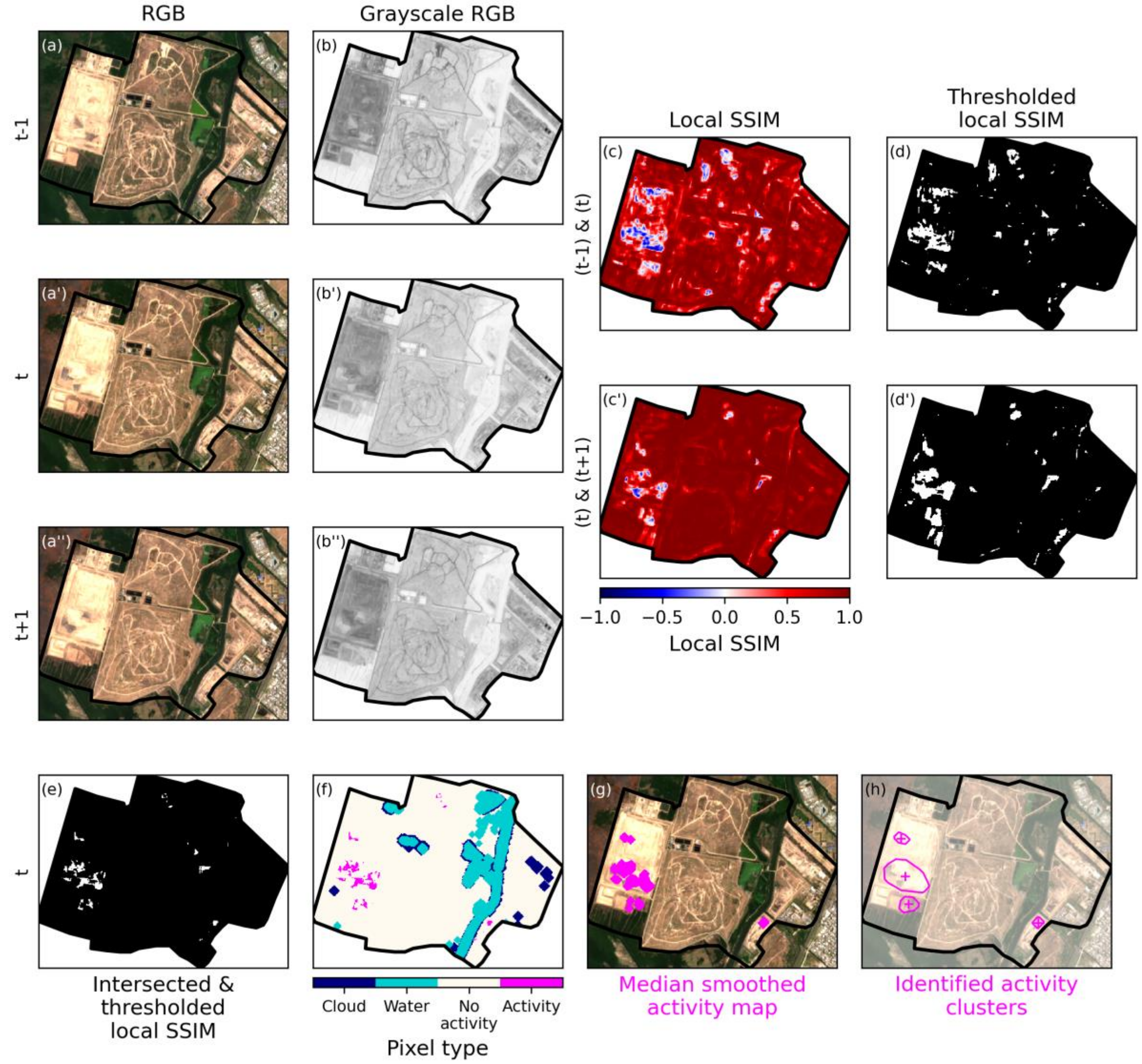

Supplementary Figure 1. **Illustration of the activity detection algorithm applied on Sentinel-2 imagery for Norte III landfill in Buenos Aires, Argentina**

RGB images for timestamps t-1, t and t+1 (a, a', a'') are converted to Grayscale RGB images (b, b', b''). Local Structural SIMilarity (SSIM) maps are computed between timestamps t-1 and t (c) and t and t+1 (c'), and strongly dissimilar areas (low SSIM values) are selected by applying a threshold (d, d'). The overlap between these two images shows current activity at timestamp t (e). Pixels that are associated with water or clouds are removed from the analysis (f), and the resulting activity map is smoothed using a median filter and dilated back afterwards (g). Spatially consistent activity clusters remain and are outlined using convex hulls (h). All this analysis is performed within landfill boundaries, outlined by the thick black line in all panels.

Supplementary Methods 2: Comparison of GHGSat-based plume sources and Sentinel-2 detected surface activity

This supplement describes the metric and method used to evaluate the proximity between GHGSat-based plume sources and Sentinel-2 detected surface activity.

For all 107 waste disposal sites with sufficient Sentinel-2 data and adequate surface activity detection results, we compare the manually verified locations of GHGSat-detected plume sources with the closest in-time Sentinel-2 image for which surface activity has been detected. The comparison process and significance metric are illustrated in Supplementary Figure 2.

For each GHGSat-detected plume source i, we compute its distance d_i to the spatially closest Sentinel-2 surface activity cluster detected in the temporally closest Sentinel-2 image (panels 1 – 14, dashed thin vertical lines in the bottom right panel of Supplementary Figure 2). We then compute the averaged distance to the closest surface activity cluster across all site-wise sources $\bar{d} = \left(\frac{1}{N}\right)\sum_{i=1}^{N} d_i$ (dashed thick vertical line in the bottom right panel of Supplementary Figure 2).

To evaluate the statistical significance of this averaged distance $\bar{d}$, for each distance d_i, we also compute the distance to the closest activity cluster d_i' distribution for 10000 points randomly drawn within the landfill mask (thin histogram lines in the bottom right panel of Supplementary Figure 2). We then compute the distribution of similarly averaged distances to the closest surface activity cluster across all site-wise random sources $\bar{d}' = \left(\frac{1}{N}\right)\sum_{i=1}^{N} d_i'$ (thick black histogram line in the bottom right panel of Supplementary Figure 2).

We finally compute the p-value, representing here the probability of obtaining averaged randomly drawn distances to the closest activity clusters smaller than what is obtained with GHGSat observations (probability of the null hypothesis yielding a result as extreme as the observations)

$$\text{p-value} = P\left(\overline{d'} \leq \bar{d}\right)$$

We consider that we find a statistically significant proximity between GHGSat plume sources and Sentinel-2 detected surface activity if p-value<0.05.

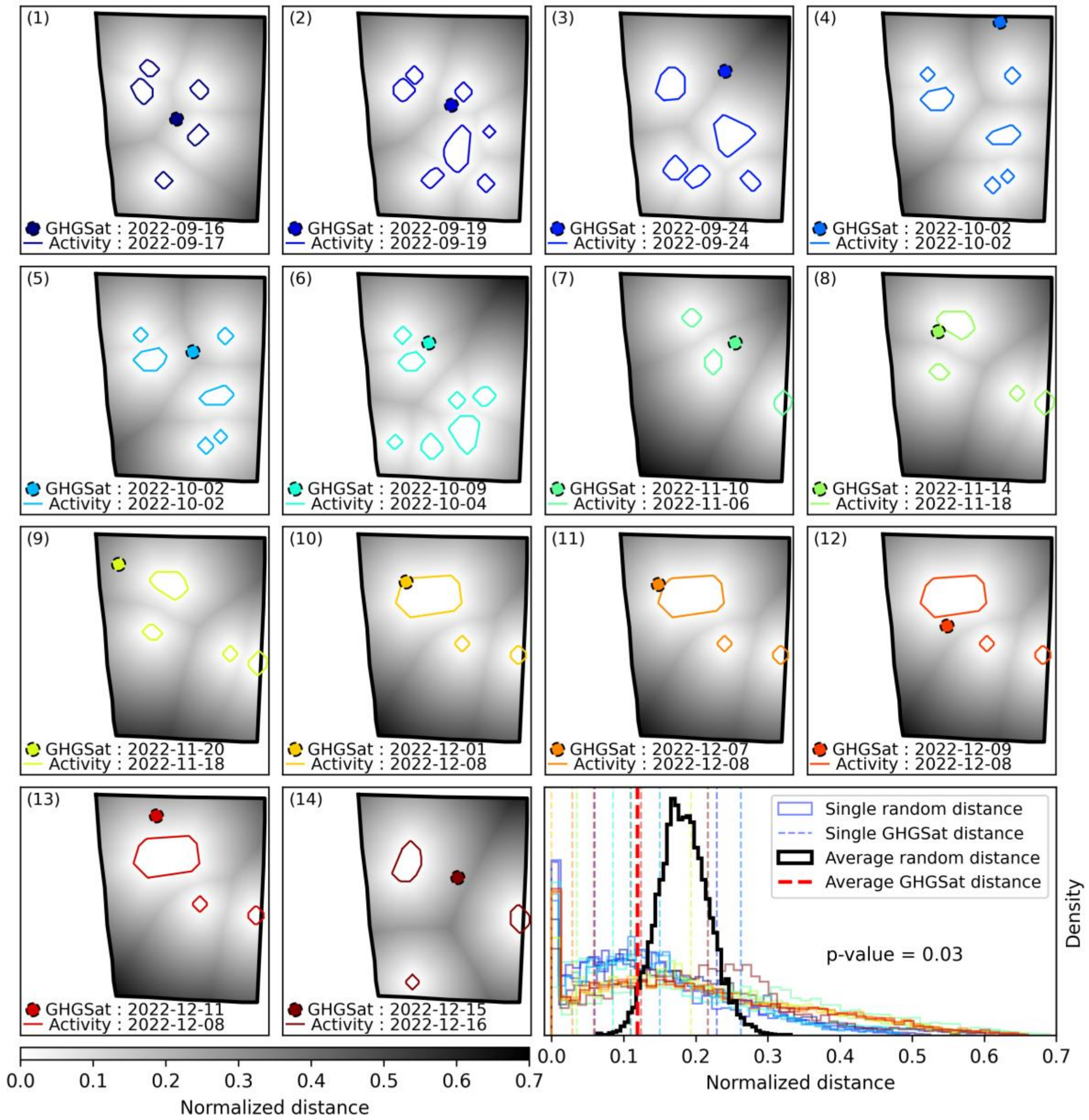

Supplementary Figure 2. Illustration of the comparison between GHGSat-detected plume sources and Sentinel-2 detected landfill surface activity over the Amman landfill, Jordan
Comparison of GHGSat-detected plume origins (color-filled dashed circles) with closest-in-time Sentinel-2 detected surface activity (colored thin full-lines) and spatial distribution of the distance to closest activity cluster (panels 1 – 14). Single and averaged distances to the closest activity-cluster for GHGSat detected plume sources (thin and thick dashed lines) and single and averaged distance to the closest activity-cluster distributions for randomly drawn plume sources (full thin and thick histogram lines, bottom right panel). All distances are normalized by the square root of the landfill's area.

Supplementary Note 1: Discussion of plume-based IME estimates of emission rates against atmospheric inversion results

For four strongly-emitting cities, Supplementary Figure 3 compares averaged plume-based IME emission estimates and posterior emission rates obtained through atmospheric inversion. The former are based on the automated TROPOMI plume detections over years 2021 and 2022 gathered for this work, and the latter are results for the year 2020 drawn from Maasakkers et al. (2022)[5]. The IME plume-based estimates show a positive bias as they only comprise data from days where emissions were high enough to allow for a plume detection, and lower emitting days that would have been accounted for in a comprehensive atmospheric inversion are not considered in this plume-based approach. This is why we consider that our plume-based IME estimates is more representative of the emission upper boundary rather than the actual average.

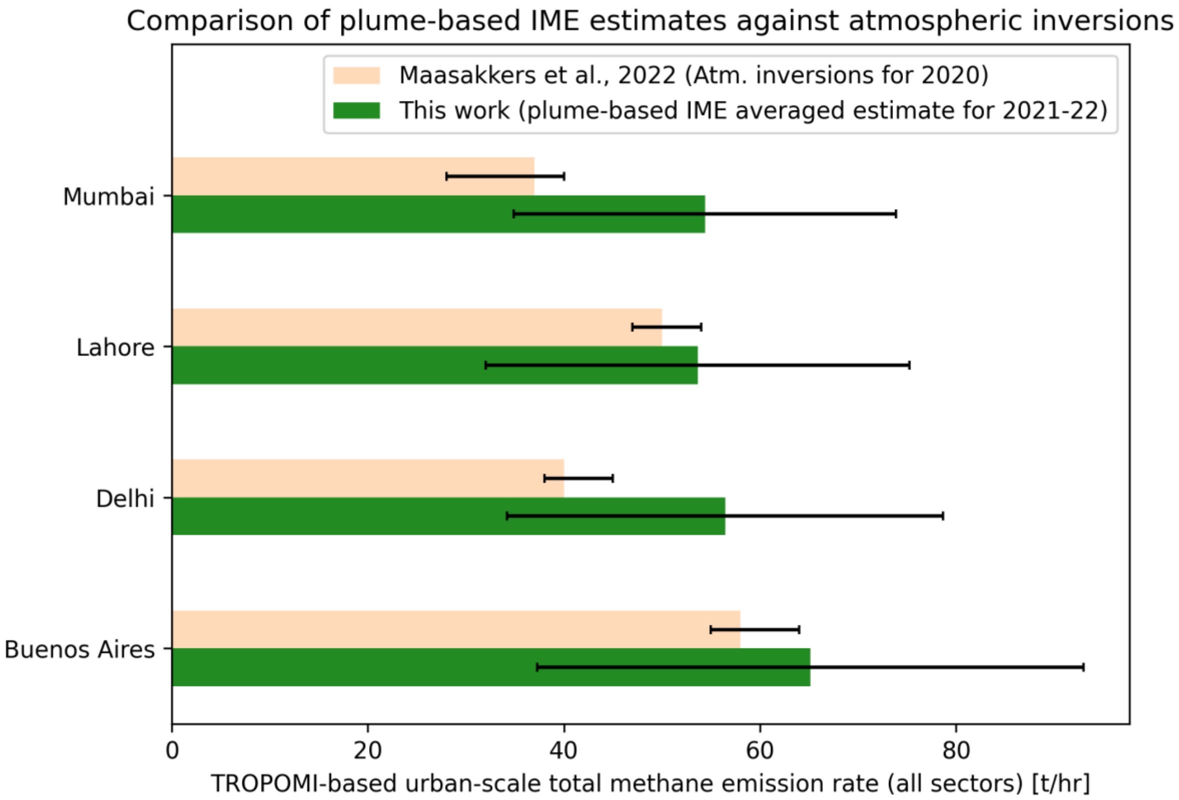

Supplementary Figure 3. Comparison of plume mass-balance and atmospheric inversion emission results

Comparison of plume-based IME estimates of total methane emission rates for four urban areas over 2021-2022 (this work) against atmospheric inversion results obtained for the same areas for 2020[5].

Supplementary Note 2: Supplementary visuals, information and data on TROPOMI-based total urban-scale methane emissions

This Supplementary Note provides additional information on TROPOMI plume detections for the urban areas targeted by GHGSat. The median number of plume detections over two years is 6 (Extended Data Figure 1). A small fraction of urban areas account for the majority of detected plumes: 14 urban areas show at least 21 detected plumes with a total encompassing 82% of all TROPOMI plumes detected for the 46 urban areas. These cities combine regular coverage with large emissions, leading to a high number of detected emission plumes. The cities are (sorted per country, themselves in alphabetical order):

- Argentina: Buenos Aires
- Bangladesh: Dhaka
- India: Delhi, Ahmedabad, Lucknow, Kanpur, Hyderabad, Mumbai, Kolkata
- Iran: Tehran
- Morocco: Casablanca
- Pakistan: Lahore, Karachi
- Spain: Madrid

Not detecting urban scale emission plumes in TROPOMI data does not mean that there are no emissions (see next Supplementary Note 3). We find that 62 urban areas do not show detected plumes because of coverage-related challenges (e.g. persistent cloudiness or sharp elevation gradients), 19 urban areas are not expected to have total emissions exceeding the ~8 t/hr TROPOMI plume detection threshold[4] based on emission inventories[2] and GHGSat estimates, and finally 3 urban areas are surrounded by artefact-causing surface albedo features, which complicate the detection of methane plumes.

Supplementary Figure 4 provides the averaged plume-based IME emission rate estimates for all the 46 urban areas that show at least one plume detection over 2021-2022. These rates have a 19 t/hr median, with 5th and 95th percentiles of 5 t/hr and 74 t/hr, respectively. Supplementary Table 1 provides TROPOMI plume-based emission rates for all urban areas that show at least one detected plume.

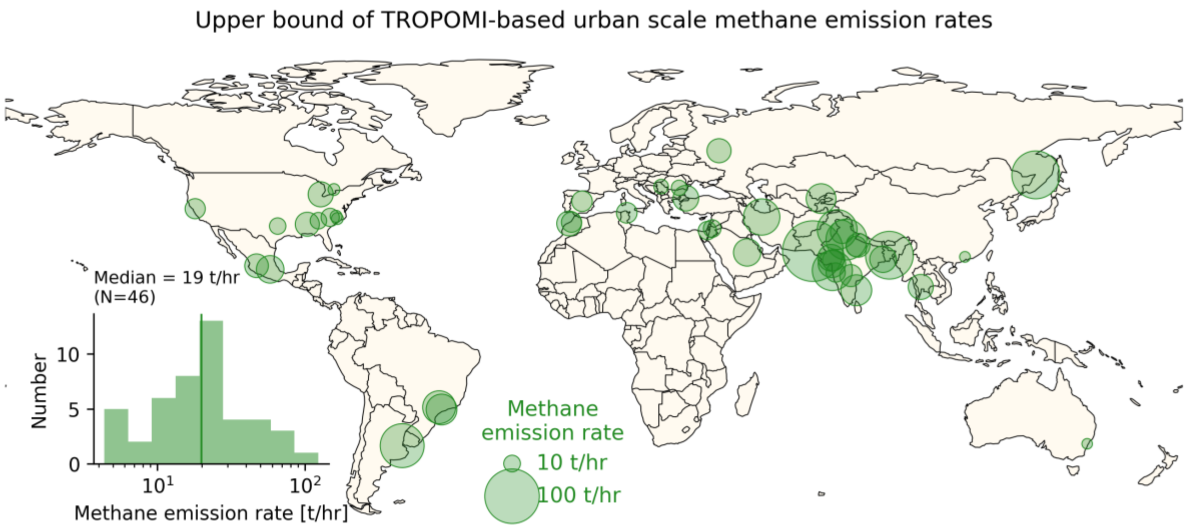

Supplementary Figure 4. Map of TROPOMI-detected urban-scale emissions
Spatial distribution of mean plume-based TROPOMI IME estimates of methane emission rate for the 46 urban areas targeted by GHGSat that show methane emission plumes in 2021-2022 TROPOMI data. As shown in Supplementary Figure 3 for four cities, IME-based estimates relying on detected plumes provide an upper bound to actual average annual emissions.

Latitude	Longitude	Country	City	N	N filtered	Q	Q_{uncert}
° North	° East					t/hr	t/hr
20.5445	-103.1738	Mexico	Guadalajara	16	15	19.38	5.66
-34.5277	-58.6243	Argentina	Buenos Aires	87	85	65.15	27.86
-33.9544	150.8649	Australia	Sydney	1	1	4.38	1.55
23.7595	90.3763	Bangladesh	Dhaka	114	108	76.73	41.02
-23.4618	-46.5848	Brazil	São Paulo	3	3	32.34	10.54
-22.7509	-47.3149	Brazil	Campinas	1	1	36.88	20.83
43.8671	-79.5011	Canada	Toronto	1	1	5.38	3.94
23.2638	113.4822	China	Guangzhou	1	1	4.54	2.28
40.2925	-3.6102	Spain	Madrid	44	41	14.75	8.06
22.9826	72.5689	India	Ahmedabad	55	52	24.69	14.49
28.5694	77.2343	India	Delhi	108	102	56.43	22.23
21.1070	72.8056	India	Surat	9	9	25.75	4.97
22.2330	73.2068	India	Vadodara	5	5	16.16	5.11
22.5498	88.4343	India	Kolkata	26	25	24.19	12.68
19.0972	72.9412	India	Mumbai	26	24	54.39	19.50
18.4715	73.9509	India	Pune	6	6	13.09	3.28
30.9293	75.9071	India	Ludhiana	10	9	18.88	8.24
17.5181	78.5931	India	Hyderabad	27	25	17.00	6.76
13.0441	80.2461	India	Chennai	4	4	32.59	12.55
26.4490	80.2346	India	Kanpur	27	25	14.57	7.56
26.7970	80.7815	India	Lucknow	36	35	19.77	15.54
35.4587	51.3319	Iran	Tehran	21	20	44.69	10.35
31.3194	34.7371	Israel	Be'er Sheva	3	3	11.92	5.26
31.9312	36.1853	Jordan	Amman	9	8	11.06	3.90
33.4822	-7.5390	Morocco	Casablanca	25	24	21.29	6.51
33.8708	-6.8108	Morocco	Rabat	2	2	14.33	5.00

19.4399	-99.0060	Mexico	Mexico City	6	6	26.11	3.53
31.6273	74.4183	Pakistan	Lahore	93	88	53.64	21.60
25.0198	66.9782	Pakistan	Karachi	43	42	123.41	43.72
44.3968	26.0568	Romania	Bucharest	6	6	8.53	3.02
48.3639	135.1516	Russia	Khabarovsk	1	1	77.41	35.71
55.7535	38.2882	Russia	Moscow	2	2	20.15	4.11
24.6161	46.8914	Saudi Arabia	Riyadh	15	15	25.80	11.16
44.7848	20.5944	Serbia	Belgrade	2	2	7.75	2.05
14.0590	99.9718	Thailand	Bangkok	2	2	21.68	5.20
36.7379	10.0777	Tunisia	Tunis	2	2	14.88	1.72
41.2151	28.1497	Turkey	Istanbul	4	4	22.69	11.20
33.2430	-87.6518	United States	Tuscaloosa	3	3	20.05	1.93
37.9970	-121.9371	United States	Oakland	2	2	14.28	7.14
34.3274	-84.2442	United States	Atlanta	7	7	9.83	3.20
42.4023	-83.5555	United States	Detroit	1	1	21.76	9.87
35.3412	-80.6565	United States	Charlotte	18	18	11.78	5.92
34.9800	-78.4621	United States	Fayetteville	3	3	5.33	1.01
35.6757	-78.8478	United States	Raleigh	1	1	5.44	2.47
32.6824	-96.7085	United States	Dallas	1	1	9.73	7.99
41.0976	69.4832	Uzbekistan	Tashkent	18	17	30.29	17.73

Supplementary Table 1. Summary of TROPOMI-based data and emission estimates

Summary of TROPOMI-based results for all urban areas that show at least one plume detection in 2021-2022 TROPOMI data. 'N' and 'N filtered' denote the number of detections obtained without and with filtering emission rates at 2σ. Q denotes the averaged TROPOMI plume-based emission rate, and Q_{uncert} the uncertainty.

Supplementary Note 3: Explanations on TROPOMI urban hotspot coverage

The 151 landfills observed by GHGSat are located in 130 different urban areas, the relevant scale at which TROPOMI can resolve emissions. Out of those 130 urban areas, 46 show at least one manually-verified methane emission plume detection in TROPOMI data. Thus, most of targeted urban areas do not feature any detected TROPOMI methane plumes, which we explore in this Supplement.

For that purpose, we designed some metrics computed from the TROPOMI and GHGSat data, and the EDGAR emission inventory[2] to characterize each of these urban areas in terms of data coverage, surface roughness, albedo, and expected methane emissions. Supplementary Table 2 describes these metrics. We determined empirical thresholds and boolean operations to label and list all the different reasons that can explain why TROPOMI data may not show any methane emission plume at a given location. Supplementary Table 3 lists the boolean operations and thresholds used to label each urban area using our metrics. These thresholds do not perfectly separate urban areas with methane plumes from the ones with no detected plumes, but they draw a fair boundary between these two categories, also realizing the number of plume detections at a given location can vary due to different meteorological parameters (e.g. cloud cover, wind speed, etc).

Metric name	Definition
E_{GHGSat}	Total GHGSat-observed methane emission rate for the landfills included in the urban area, in t/hr
E_{EDGAR}	Total urban area methane emissions as reported in EDGAR v8 (all sectors) integrated over a 50 km radius from any GHGSat-observed landfill included in the urban area, in t/hr
C	Maximum number of TROPOMI overpasses with valid observations in 2 years (2021-2022) contained within a 0.3°x0.3° square centered on the considered landfill observed by GHGSat in the urban area, computed as a percentage of 730.5 observations (1 per day). This metric measures the overall coverage of TROPOMI over the urban area.
W_{small}	Fraction of 0.01°x0.01° oversampled pixels that show less than 75 valid observations over 2 years (2021-2022) within a 0.3°x0.3° image centered on the main landfill observed by GHGSat in the urban area. This metric assesses the area covered by waterbodies, sharp elevation changes and persistent cloudiness hampering TROPOMI coverage in the urban area.
W_{large}	Same as W_{small} but considering a 2°x2° image centered on the main landfill to assess coverage around the urban area.
R_{corr}	Pearson correlation coefficient between 0.3°x0.3° images oversampled at a resolution of 0.01°x0.01° of 2-year (2021-2022) averaged surface reflectance near 2.3 µm and 2-year average methane total column. This metric measures to what extent spatial albedo features may cause albedo-related artefacts in methane observations over the urban area.

Supplementary Table 2. Definition of urban hotspot metrics related to TROPOMI data coverage

Metrics designed to characterize each urban hotspot in terms of expected methane emissions and TROPOMI data coverage and surface albedo.

Possible reason explaining why no TROPOMI plume is detected	Boolean operation to verify
Low mean coverage	$(C < 30)$
Missing data (due to water bodies, sharp elevation changes, persistent cloudiness, etc.)	$(W_{small} > 0.2)$ OR $(W_{large} > 0.4)$
Correlated albedo	$\lvert R_{corr} \rvert > 0.9$
Low emissions	($E_{GHGSat} < 2$ t/hr) OR ($E_{EDGAR} < 8$ t/hr)

Supplementary Table 3. Metric thresholds used to explain the absence of TROPOMI plume detection

Boolean operations that determine which possible reasons explain that no TROPOMI plume is detected for a given urban area.

For the 84 urban areas that do not show any plume in TROPOMI data, we only report the primary reason possibly explaining why, following the order of Supplementary Table 3. Results are shown in Extended Data Figure 2. We find that the absence of a detected plume can be explained by coverage-related issues for (26 + 36 =) 62 urban areas. These coverage issues can be persistent, due to waterbodies or sharp elevation changes near these urban areas, or depend on climate and meteorology with frequent cloudiness. Besides, for 3 urban areas, plume detections are hampered by sharp changes in surface albedo that cause methane total column retrieval errors correlated to albedo variations. These scenes are automatically rejected by our machine learning scheme as well[4]. Finally, given that TROPOMI has an overall 8 t/hr plume detection threshold[4], we find that low expected methane emissions explain the remaining urban areas where we do not detect any methane plume in TROPOMI data (N=19).

Supplementary Note 4: Supplementary visuals and information on GHGSat-based facility-scale methane emissions

This supplement provides additional information on the set of GHGSat observations used in this work. Supplementary Figure 5 shows the site-wise detection frequency. Supplementary Figure 6 gives the distributions of the single-plume emission rates, their associated uncertainties and draws the relationship between wind speed and single-plume emission rate uncertainty. Supplementary Table 4 summarizes our results at site level. Finally, Extended Data Figure 7 is discussed, comparing the GHGSat detection limit to the cumulative emission and site number distribution functions of other facility-scale bottom-up datasets, illustrating the potential of high-resolution satellite observations to cover most of the total facility-scale solid waste methane emissions.

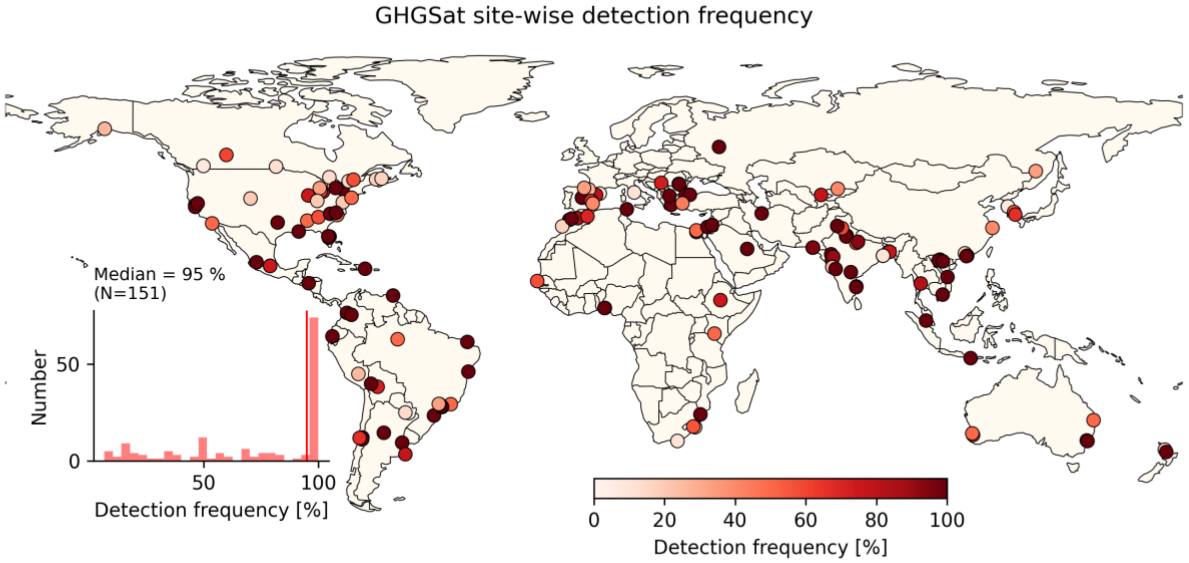

Supplementary Figure 5. Map of GHGSat site-wise positive emission detection frequency
Spatial and site-wise detection frequency distributions of the 151 waste-disposal sites observed by GHGSat satellites. All sites have at least one plume detection.

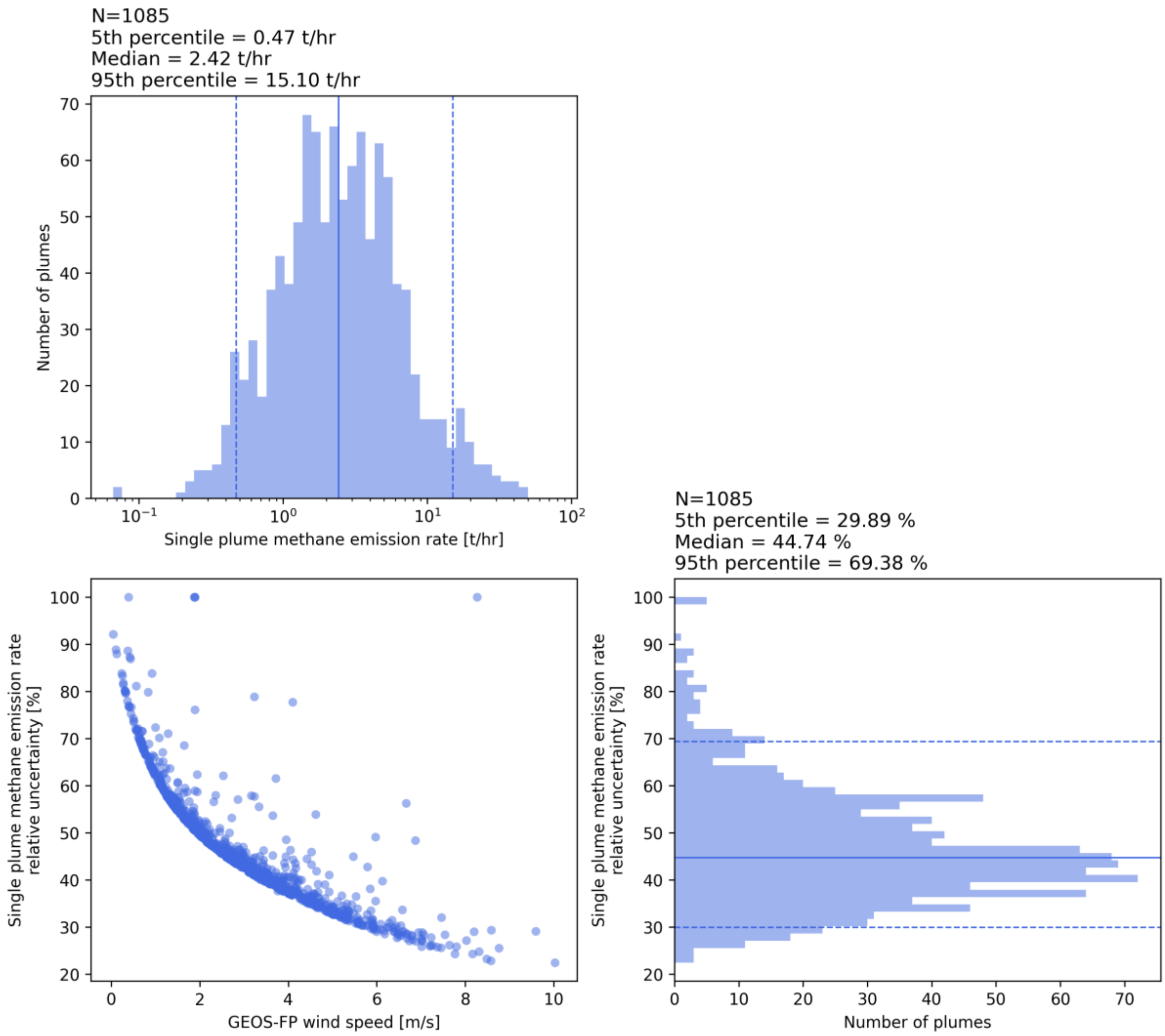

Supplementary Figure 6. GHGSat single plume emission rate and uncertainty distributions
GHGSat single plume methane emission rate (top left) and relative uncertainty (bottom right) distributions. The relationship between wind speed and the relative uncertainty for single plume emission rate is also provided (bottom left).

Discussion on emission rate distributions in facility-scale bottom-up databases

The Climate TRACE dataset[42] is a global scale facility-level emission dataset that includes both managed landfills and dumping sites. As illustrated in Extended Data Figure 7, it shows a bend in its total emission and total number of sites cumulative distributions around 2.5 t/hr, where the few sites above this threshold (~1% of total site number) represent ~12.5% of total Climate TRACE emissions (~36.7 Mt/yr, EDGAR v8 includes a total of 38 Mt/yr). The largest contribution to total emissions comes from sites emitting between 0.1 and 2.5 t/hr. Overall, sites emitting above 0.1 t/hr amount to 54% of all Climate TRACE sites while gathering 96% of total Climate TRACE emissions. Similarly, in reported datasets, sites emitting above 0.1 t/hr account for 97%, 93% and 77% of total reported emission for the US GHGRP, Canada GHGRP and European E-PRTR, respectively. These high coverage ratios highlight the potential of high-resolution satellite imagery to monitor solid waste methane emission globally (for sites with localized enough emission sources).

Site ID.	Lat	Lon	Country	N	Years	Q	Q_{unc}	Q_{RP}	Q_{CT}
	° N	° E				t/hr	t/hr	t/hr	t/hr
1	20.5445	-103.1738	Mexico	10	2022	2.40	0.73	-	11.82
2	32.4107	-116.7465	Mexico	2	2022	0.75	0.61	-	9.26
3	42.9284	-76.8487	United States	5	2022	4.21	1.32	0.74	0.73
4	-34.5277	-58.6243	Argentina	67	2021-22	21.99	1.92	-	3.23
5	-31.5172	-64.2325	Argentina	3	2022	2.16	0.57	-	1.70
6	-38.0676	-57.6486	Argentina	32	2021-22	1.06	0.15	-	1.05
7	-33.8639	150.7606	Australia	1	2022	1.69	1.02	-	1.19
8	-27.6621	152.8280	Australia	4	2022	0.46	0.31	-	1.15
9	-34.0449	150.9692	Australia	1	2022	3.72	1.81	-	1.17
10	-32.1958	115.9654	Australia	3	2022	0.22	0.13	-	0.01
11	-31.7057	115.7253	Australia	2	2022	0.18	0.16	-	1.27
12	23.7985	90.3013	Bangladesh	10	2021-22	4.56	0.97	-	1.54
13	23.7206	90.4513	Bangladesh	22	2021-22	2.22	0.49	-	-
14	-17.4766	-66.1273	Bolivia	11	2021-22	0.56	0.17	-	1.69
15	-16.5560	-68.1284	Bolivia	1	2022	1.26	0.61	-	1.76
16	-2.9551	-60.0121	Brazil	2	2022	1.48	1.27	-	-
17	-12.8558	-38.3734	Brazil	5	2022	1.67	0.31	-	1.85
18	-3.7953	-38.6823	Brazil	2	2022	3.13	0.85	-	5.80
19	-22.7930	-43.7556	Brazil	2	2022	2.41	1.97	-	-
20	-23.3450	-46.7718	Brazil	13	2021-22	4.32	0.80	-	-
21	-26.2496	-48.9024	Brazil	1	2022	0.71	0.44	-	-
22	-23.6368	-46.4220	Brazil	1	2022	4.14	1.86	-	1.75
23	-23.4037	-46.5605	Brazil	2	2022	3.53	1.26	-	-
24	-22.7784	-47.2058	Brazil	2	2022	5.80	3.49	-	-
25	-22.7234	-47.4239	Brazil	3	2022	0.14	0.14	-	1.65
26	53.3062	-112.3980	Canada	5	2021-22	0.33	0.15	0.11	-
27	49.9581	-119.4192	Canada	12	2022	0.03	0.04	-	-
28	49.7648	-97.1911	Canada	16	2021-22	0.12	0.09	2.19	1.98
29	45.8828	-66.6077	Canada	6	2022	0.13	0.16	0.36	0.34
30	46.0910	-64.9114	Canada	6	2021-22	0.09	0.11	0.47	-
31	42.0887	-82.8659	Canada	6	2021-22	1.50	0.45	1.28	1.25
32	43.8671	-79.5011	Canada	6	2021-22	0.20	0.19	2.15	2.16
33	46.5044	-80.9016	Canada	7	2022	0.06	0.07	-	-
34	45.3122	-74.9977	Canada	7	2021-22	0.08	0.09	0.42	-
35	45.7484	-73.5377	Canada	14	2021-22	0.72	0.23	0.16	-
36	-32.9521	-70.8010	Chile	1	2022	6.57	2.27	-	11.82
37	-33.5249	-70.8703	Chile	1	2022	2.79	1.31	-	3.14
38	-33.0898	-71.6372	Chile	3	2022	1.22	0.60	-	0.83
39	23.2638	113.4822	China	6	2021-22	1.07	1.12	-	10.17
40	22.5451	114.1701	China	12	2021-22	9.99	2.66	-	3.74
41	22.4176	113.9332	China	17	2021-22	8.07	1.30	-	8.47
42	31.0521	121.8871	China	20	2021-22	1.06	0.35	-	-
43	5.0812	-75.5047	Colombia	1	2022	0.65	0.37	-	1.73
44	4.4931	-74.1456	Colombia	2	2022	2.26	1.23	-	1.77

45	18.5618	-69.9699	Dominican Republic	3	2022	3.28	1.03	-	1.78
46	-2.0726	-79.9611	Ecuador	4	2022	3.02	1.17	-	-
47	29.7953	31.3558	Egypt	2	2022	2.33	0.64	-	-
48	30.2801	31.3582	Egypt	4	2022	0.40	0.29	-	3.26
49	41.5211	1.8066	Spain	12	2021-22	0.40	0.44	0.24	0.14
50	41.0783	0.6907	Spain	5	2022	0.68	0.28	-	-
51	40.2632	-3.6305	Spain	35	2021-22	4.49	0.58	0.79	1.55
52	40.3219	-3.5899	Spain	31	2021-22	2.97	0.42	0.25	0.33
53	42.7726	-1.5493	Spain	4	2022	0.32	0.31	0.10	0.10
54	43.2382	-2.9693	Spain	3	2022	0.60	0.58	-	0.12
55	39.2832	-0.8672	Spain	6	2022	1.24	0.49	0.16	0.14
56	38.4865	-0.3394	Spain	5	2022	0.75	0.48	-	-
57	8.9776	38.7121	Ethiopia	12	2021-22	1.48	0.36	-	0.95
58	38.0722	23.6496	Greece	9	2022	4.18	0.84	-	10.18
59	40.8490	23.0803	Greece	5	2022	2.93	0.97	0.68	0.61
60	14.1494	-87.2238	Honduras	1	2021	0.64	0.41	-	2.06
61	-8.7225	115.2222	Indonesia	1	2022	1.89	0.62	-	0.99
62	22.9826	72.5689	India	25	2021-22	3.62	0.37	-	2.20
63	28.7428	77.1552	India	2	2022	1.29	0.43	-	1.38
64	28.6227	77.3260	India	32	2021-22	2.53	0.26	-	1.95
65	28.5096	77.2841	India	2	2021	2.21	0.74	-	1.93
66	21.1070	72.8056	India	25	2021-22	2.89	0.36	-	0.17
67	22.2330	73.2068	India	22	2022	0.83	0.13	-	0.35
68	28.4026	77.1719	India	10	2021-22	1.45	0.25	-	0.02
69	22.5363	88.4241	India	10	2021-22	1.69	0.39	-	2.03
70	22.5633	88.4444	India	10	2021-22	0.09	0.10	-	0.41
71	19.1241	72.9526	India	22	2021-22	9.01	1.49	-	0.44
72	19.0703	72.9298	India	16	2021-22	0.32	0.16	-	2.40
73	18.4715	73.9509	India	11	2022	0.78	0.11	-	0.28
74	30.9293	75.9071	India	5	2022	0.61	0.28	-	-
75	17.5181	78.5931	India	23	2021-22	6.39	0.75	-	-
76	13.1352	80.2678	India	2	2022	1.73	0.61	-	2.24
77	12.9530	80.2245	India	4	2022	3.51	1.16	-	2.08
78	26.4490	80.2346	India	15	2022	0.61	0.27	-	2.27
79	26.7970	80.7815	India	15	2022	1.64	0.27	-	-
80	35.4587	51.3319	Iran	1	2022	9.43	4.93	-	20.44
81	31.3194	34.7371	Israel	3	2022	3.46	1.77	-	-
82	41.8542	12.3392	Italy	19	2021-22	0.04	0.03	-	-
83	31.9312	36.1853	Jordan	13	2022	5.48	1.13	-	7.26
84	-1.2486	36.8959	Kenya	2	2022	0.88	0.70	-	1.95
85	42.9676	74.5909	Kyrgyzstan	25	2021-22	0.31	0.10	-	2.05
86	37.5754	126.5988	South Korea	30	2021-22	0.27	0.11	-	20.50
87	37.5798	126.6210	South Korea	30	2021-22	0.11	0.08	-	20.50
88	35.8749	128.5181	South Korea	2	2022	0.47	0.47	-	1.25
89	35.1252	128.8742	South Korea	3	2022	1.14	0.67	-	-
90	34.0040	-4.9351	Morocco	12	2021-22	1.66	0.47	-	1.98
91	33.4822	-7.5390	Morocco	30	2021-22	5.13	0.57	-	1.64
92	31.5206	-9.6627	Morocco	12	2021-22	0.18	0.13	-	-
93	34.5802	-1.9442	Morocco	10	2021-22	0.57	0.18	-	0.99

94	33.8708	-6.8108	Morocco	7	2021-22	3.42	0.53	-	-
95	19.4582	-99.0170	Mexico	4	2021-22	1.90	0.96	-	1.72
96	19.4216	-98.9950	Mexico	4	2021-22	1.01	0.58	-	6.88
97	-25.8999	32.5973	Mozambique	2	2022	1.05	0.42	-	0.80
98	2.7327	101.6038	Malaysia	1	2022	6.94	4.18	-	-
99	6.5963	3.3746	Nigeria	3	2021-22	1.23	0.33	-	2.02
100	6.5630	3.2531	Nigeria	4	2022	0.75	0.28	-	1.64
101	-36.6592	174.6237	New Zealand	12	2021-22	0.21	0.17	-	1.46
102	-37.3693	175.0583	New Zealand	1	2021	0.40	0.20	-	2.47
103	31.6273	74.4183	Pakistan	57	2021-22	5.71	0.78	-	-
104	25.0099	66.9236	Pakistan	25	2022	0.48	0.15	-	-
105	25.0297	67.0328	Pakistan	20	2021-22	2.96	0.46	-	1.96
106	-13.5506	-72.0169	Peru	4	2022	0.13	0.15	-	1.13
107	-25.3289	-57.6425	Paraguay	7	2021-22	0.22	0.22	-	-
108	44.4785	25.9851	Romania	3	2022	0.77	0.57	-	-
109	44.3152	26.1285	Romania	7	2022	2.14	0.57	0.05	0.05
110	48.3639	135.1516	Russia	6	2022	0.12	0.08	-	-
111	55.7535	38.2882	Russia	2	2022	3.03	0.89	-	-
112	24.6161	46.8914	Saudi Arabia	7	2022	5.22	1.34	-	1.88
113	14.8024	-17.3131	Senegal	40	2021-22	2.34	0.43	-	1.78
114	44.7848	20.5944	Serbia	11	2021-22	2.38	0.67	-	1.59
115	14.0590	99.9718	Thailand	18	2021-22	2.88	0.53	-	-
116	10.3753	-61.4098	Trinidad and Tobago	1	2022	1.19	0.58	-	-
117	36.7379	10.0777	Tunisia	9	2022	3.04	0.62	-	-
118	41.2151	28.1497	Turkey	19	2022	7.21	0.99	-	0.03
119	41.1477	29.3709	Turkey	4	2022	3.50	1.65	-	11.31
120	38.5329	27.0682	Turkey	9	2022	0.86	0.49	-	0.66
121	61.2903	-149.6054	United States	23	2021-22	0.68	0.43	0.78	0.78
122	33.2430	-87.6518	United States	6	2021-22	0.31	0.18	-	2.06
123	37.9970	-121.9371	United States	6	2021	0.30	0.23	0.57	0.57
124	37.4581	-121.9416	United States	2	2021	1.48	0.67	0.70	0.70
125	38.5213	-121.1857	United States	3	2021	0.85	0.30	0.70	0.70
126	40.0177	-105.0143	United States	5	2021-22	0.18	0.19	0.50	0.44
127	28.4000	-80.8275	United States	2	2021-22	3.06	2.55	2.75	2.53
128	28.0581	-81.0973	United States	2	2021-22	3.58	2.28	0.52	0.62
129	28.4734	-81.2225	United States	3	2022	3.38	1.77	0.76	0.33
130	34.3274	-84.2442	United States	7	2021-22	0.56	0.31	2.04	3.53
131	40.9375	-87.3380	United States	8	2021-22	1.15	0.37	0.64	0.57
132	29.9343	-90.2610	United States	2	2021	4.35	1.99	1.10	0.29
133	38.8536	-76.7885	United States	5	2021-22	0.17	0.19	0.24	0.28
134	42.4023	-83.5555	United States	2	2022	0.19	0.17	0.87	0.98
135	43.1734	-83.8347	United States	3	2021	0.35	0.33	2.31	2.31
136	35.3412	-80.6565	United States	5	2022	2.25	0.60	0.83	-
137	34.9800	-78.4621	United States	9	2021-22	0.82	0.41	0.89	3.76
138	35.6757	-78.8478	United States	2	2022	0.89	0.38	0.68	0.72
139	40.2387	-74.1144	United States	10	2021-22	0.72	0.31	0.99	1.00
140	43.2100	-78.9745	United States	4	2022	1.20	0.27	1.66	1.34
141	39.2804	-84.5965	United States	5	2021	0.20	0.20	1.05	1.05
142	32.6824	-96.7085	United States	1	2021	1.87	0.78	1.81	1.81

143	41.0976	69.4832	Uzbekistan	41	2021-22	1.75	0.28	-	-
144	16.0423	108.1423	Vietnam	2	2022	2.05	0.96	-	0.03
145	10.6675	106.6724	Vietnam	1	2022	6.12	3.10	-	-
146	21.3361	105.8344	Vietnam	2	2022	7.12	2.62	-	-
147	21.1293	105.4232	Vietnam	5	2022	2.26	0.82	-	-
148	20.8150	106.7551	Vietnam	1	2022	1.02	0.61	-	-
149	-29.8179	30.9800	South Africa	5	2021-22	0.17	0.18	-	9.04
150	-34.0175	25.5669	South Africa	30	2021-22	0.06	0.04	-	1.57
151	-29.6066	30.4185	South Africa	18	2021-22	0.55	0.25	-	2.32

Supplementary Table 4. Summary of site level GHGSat results

'Lat' and 'Lon' denote 'Latitude' and 'Longitude'; 'N' gives the number of GHGSat observations per site and 'Years' the years they cover. 'Q' and 'Q_{unc}' give the average emission rates and uncertainties, respectively. Q_{RP} provides reported emission rates in national reporting programs (if available) and Q_{CT} provides the emission rate computed by the Climate TRACE initiative (if available).

Supplementary Note 5: Analysis of possible meteorological drivers and seasonality of methane emissions from waste disposal sites

This supplement explores the possible drivers of methane emissions from waste disposal sites by meteorology and seasonality.

The Extended Data Figure 4 shows methane emission rate deviations from site-wise medians against meteorological parameter deviations from the median, for different meteorological parameters (panels (a) to (e)), and against seasons (panel (f)). All panels also include first-order sensitivity indices that describe the amount of variance explained by each parameter. We have $S_i = V_{X_i}(E_{\sim X_i}(Q|X_i))/V(Q)$, with Q, the emission rate, X_i a meteorological variable, V and V_{X_i} the usual variance and the variance across all X_i values resp. and $E_{\sim X_i}$, the expectation across all parameter values but for X_i which value is fixed. Overall, we conclude from the very low first-order sensitivity indices S_i displayed in Extended Data Figure 4 (ranging 0.01 – 0.06) that none of the explored meteorological parameters shows convincing signs of driving emissions.

This absence of meteorological driving or its undetectability may be explained by the level of single-observation quantification uncertainty. Supplementary Figure 7 compares the absolute difference in emission rate against the sum of single observation uncertainty for all available site-wise positive detection pairs. We can observe that most of the emission rate differences are lower than the sum of uncertainties, meaning that most of the variability could be explained by single observation uncertainty.

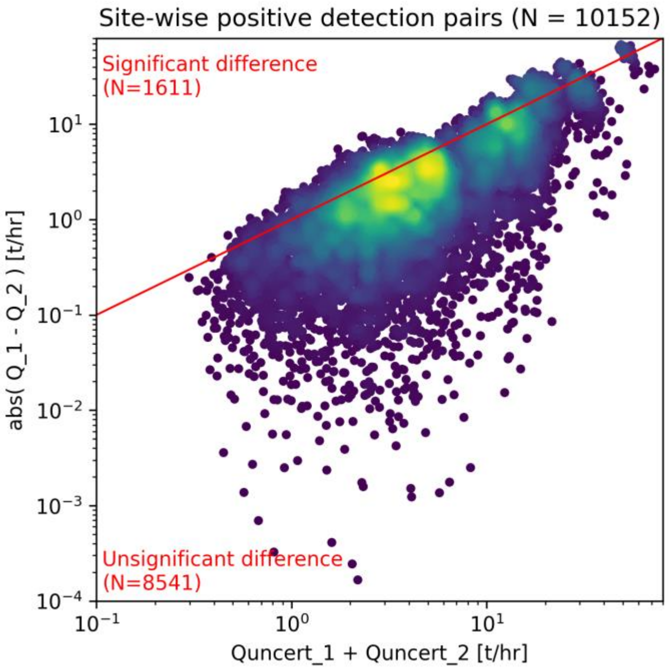

Supplementary Figure 7. Significance assessment of site-wise plume-to-plume emission difference

Absolute difference in emission rate (Q_i) compared to single observation uncertainty (Quncert_i) sum for all site-wise positive detection pairs.

Supplementary Note 6: Comparison of managed landfills and dumping site averaged site-wise emission rate distributions

This supplement provides a comparison of site-wise averaged emission rate distributions for managed landfills and dumping sites. First, Supplementary Figure 8 compares raw averaged emission rates and no-detection frequency between managed landfill and dumping sites. We perform two different statistical tests to assess whether the samples for managed landfills and dumping sites follow similar underlying distributions: a two-sided two-sample Kolmogorov-Smirnov (K-S) and a two-sample Anderson-Darling (A-D) tests. The latter gives more weight to the tail of the distribution than the former. Here, both yield non-significant p-values. This means that the null hypothesis cannot be rejected and thus that GHGSat-based methane emission rate distributions for managed landfills and dumping sites are not significantly different.

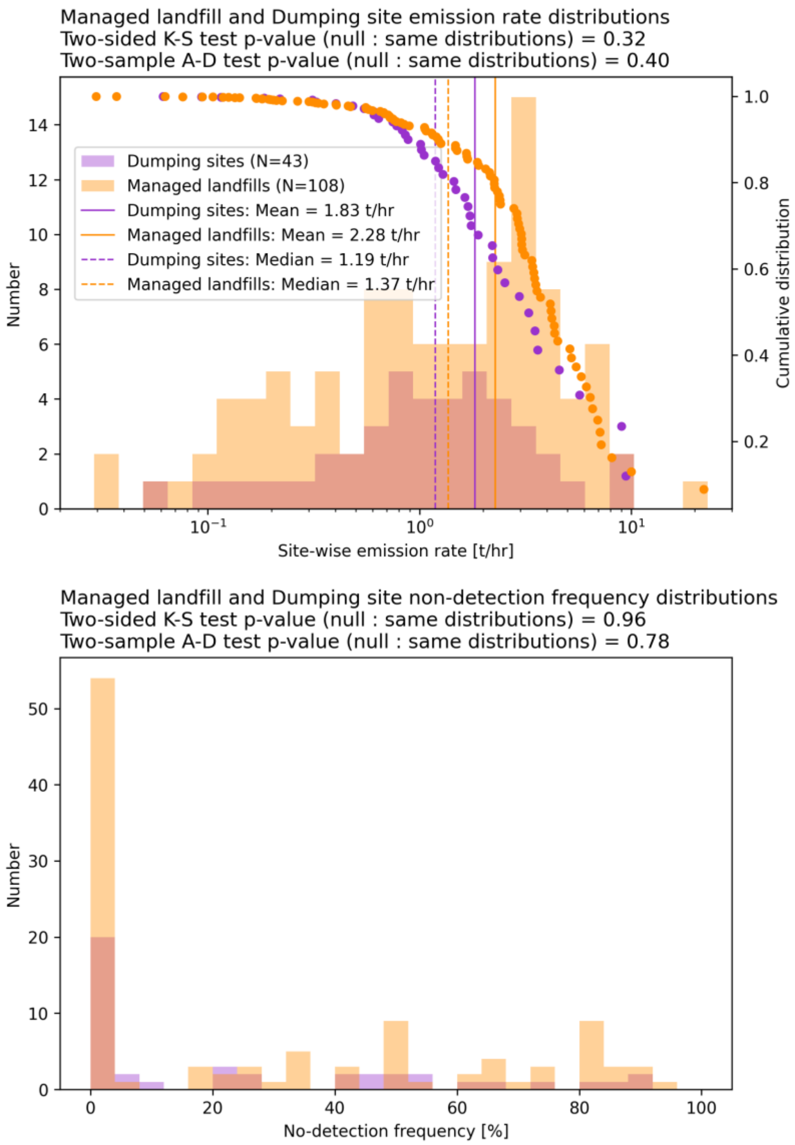

Supplementary Figure 8. Comparison of managed landfill and dumping site emission and no-detection frequency distributions

Comparison of site-wise emission (top) and no-detection frequency (bottom) distributions between managed landfills (orange) and dumping sites (purple).

We perform this comparison in an additional configuration to assess the impact of accounting for the total waste disposal site size when comparing managed landfill and dumping site emission rate distributions. The Extended Data Figure 6 showcases the same comparison as in the top panel of Supplementary Figure 8, but for emission rates per km^2, using the total waste disposal site area (determined from Google Earth imagery as detailed in Supplementary Methods 1). Considering the low p-value (0.01) by both the K-S and A-D tests for emission per area using total site area as reference, we conclude that emission per area distributions are not comparable between managed landfills and dumping sites, with dumping sites showing significantly higher emissions per area. This may be explained by the fact that managed landfills include closed inactive modules that generally show no emissions above the GHGSat detection threshold but still add to the total site area whereas, by definition, dumping sites do not show these closed inactive modules. This reflects the expected effect of definitively covering and closing some parts of managed landfills, thus confirming the efficiency of this mitigation strategy. However, managing a landfill does not mean that emissions are completely mitigated: we observe emissions arising from the open active modules from managed landfills (See Supplementary Note 10). These emissions of the active module are not fully mitigated despite the landfill being managed.

We further evaluate this comparison of (total or area-normalized) emission rate distributions between managed landfills and dumping sites by assessing the results' robustness to (1) the site type classification between managed landfills and dumping sites; and (2) to the reported site emission uncertainties.

First, to discuss the impact of site type classification, we perform the same analysis using only the sites for which Climate TRACE provides a type, either "Sanitary Landfills" or "Dumpsites" (N=88). Their classification is completely independent of ours but agrees with ours as Supplementary Figure 9 shows. Supplementary Figure 10 is similar to Extended Data Figure 6 but uses Climate TRACE's site type classification and shows that the conclusions do not change when using the different site classification.

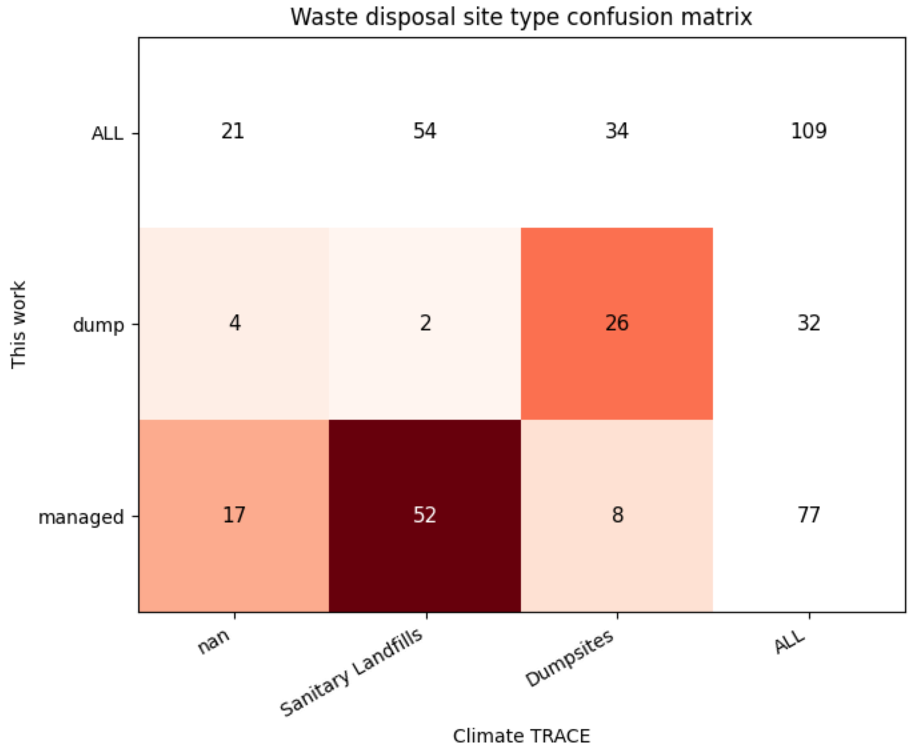

Supplementary Figure 9. Waste disposal site type confusion matrix between the Climate TRACE dataset and our own site type classification

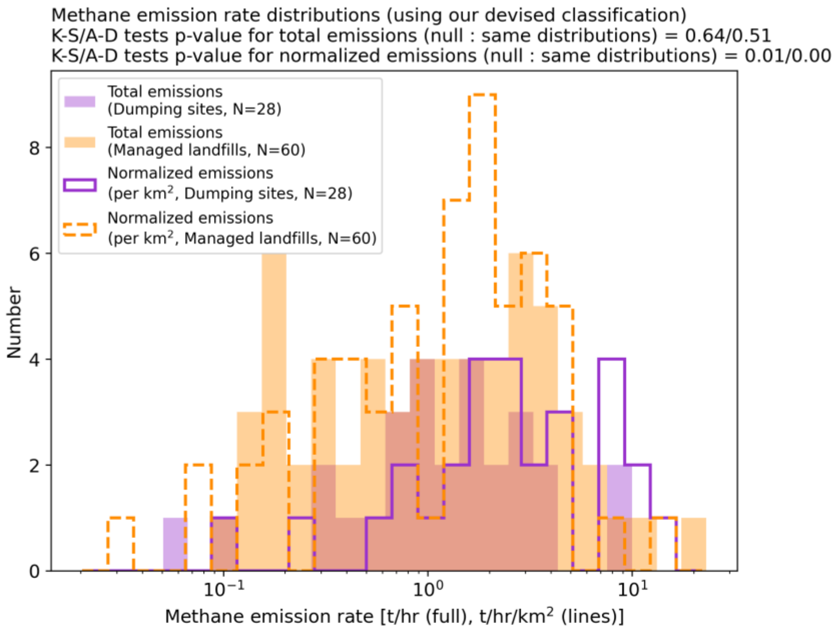

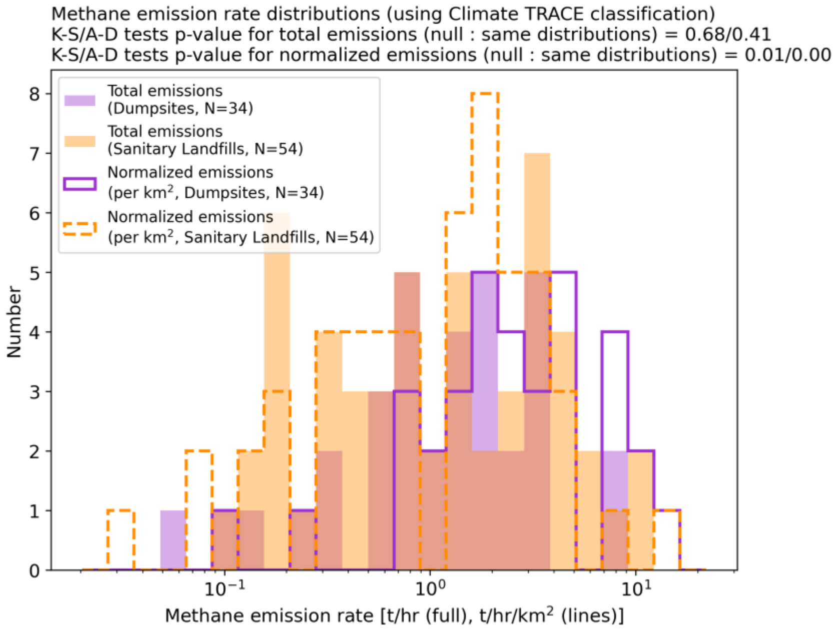

Supplementary Figure 10. **Comparison of managed landfill and dumping site total and area-normalized emission distributions for sites available in the Climate TRACE dataset**
Distributions of total (full colors) and full-area-normalized (lines) methane emission rates for "managed landfills" (orange) and "dumping sites" (purple) following our (top) and Climate TRACE's (bottom) classifications, for sites that show a non-null Climate TRACE label.

Secondly, to discuss the impact of reported site emission uncertainties, we employ a 1000 ensemble of randomly drawn emission sets for the managed landfills and the dumping sites separately, following normal distributions centered on site-wise average emission rates using site-wise emission uncertainty as standard deviation. For all 1000 randomly drawn sets of emission rates, we compare distributions for managed landfills and dumping sites for total emissions per site and emissions normalized by the total site area. Supplementary Figure 11 shows the distributions of obtained p-values for both KS and AD tests using our own site classification.

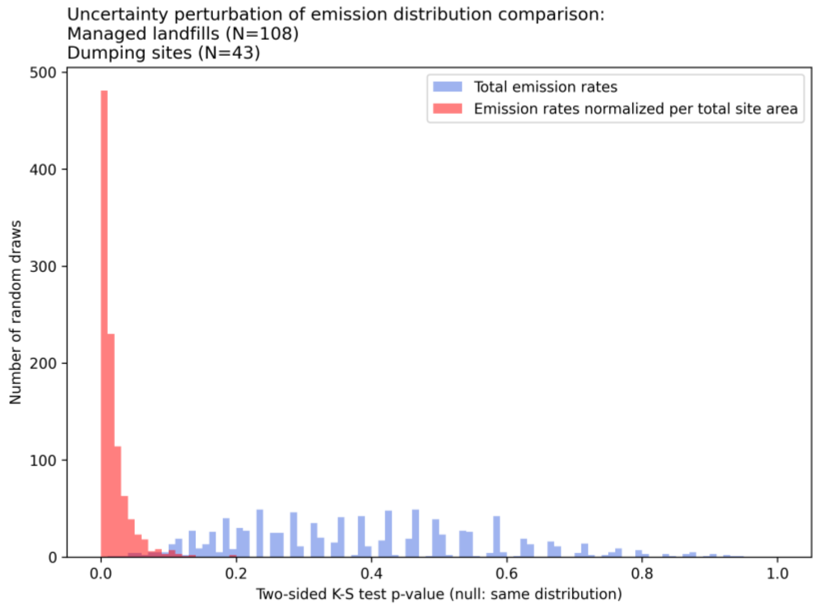

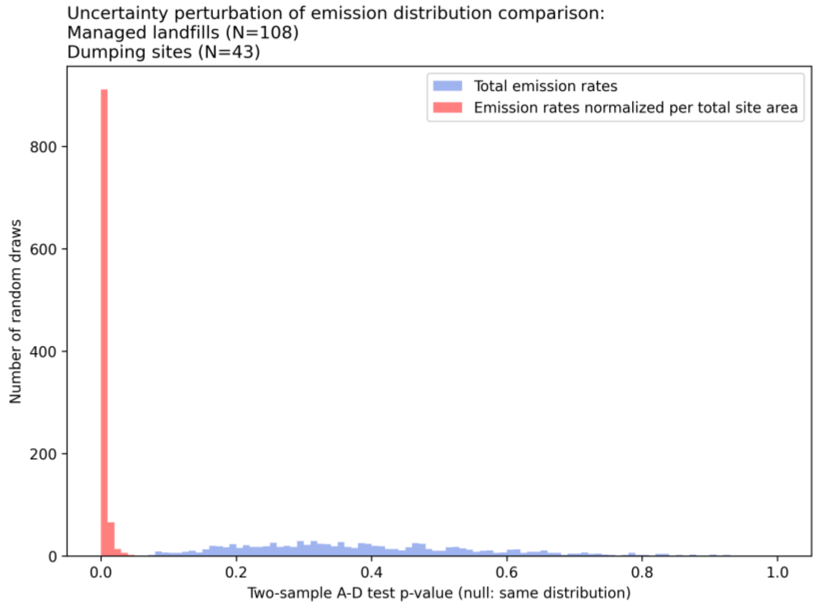

Supplementary Figure 11. Impact of site-wise emission uncertainty on managed landfill and dumping site emission distribution comparison

Distributions of p-values obtained for a two-sided Kolmogorov-Smirnov test (K-S, top) and for a two-sample Anderson-Darling test (A-D, bottom) comparing total emission rate distributions (blue) and total-area normalized emission rate distributions (red) between managed landfills and dumping sites, using our devised site classification. Low p-values mean that distribution between managed landfills and dumping sites are significantly different, while p-values larger than 0.01 mean that they are not significantly different.

P-values are overall very different from 0.01 (or lower) when comparing total emission rate distributions between managed landfills and dumping sites, and are overall close to very low values when comparing total-area normalized emission rate distributions between managed landfills and dumping sites. This means that our conclusions on differences in total or area-normalized emission rate distributions between managed landfills and dumping sites, for the sites that we observed, hold when accounting for site emission rate uncertainties.

Finally, we also assess the sensitivity of our results to the emission rate range of the sampled sites. The 2.5 t/hr inflection point in the cumulative distribution of facility-level emissions in the Climate TRACE dataset (Extended Data Figure 7) indicates that sites emitting below this threshold account for the majority of total emissions. Consequently, we also discuss our comparison of managed landfill and dumping site emissions using only sites emitting below 2.5 t/hr. Supplementary Figure 12 reproduces Extended Data Figure 6 for the 106/151 sites that emit below 2.5 t/hr. We note that our conclusions remain unchanged: total emission distributions are not significantly different between managed landfills and dumping sites, while area-normalized emissions are.

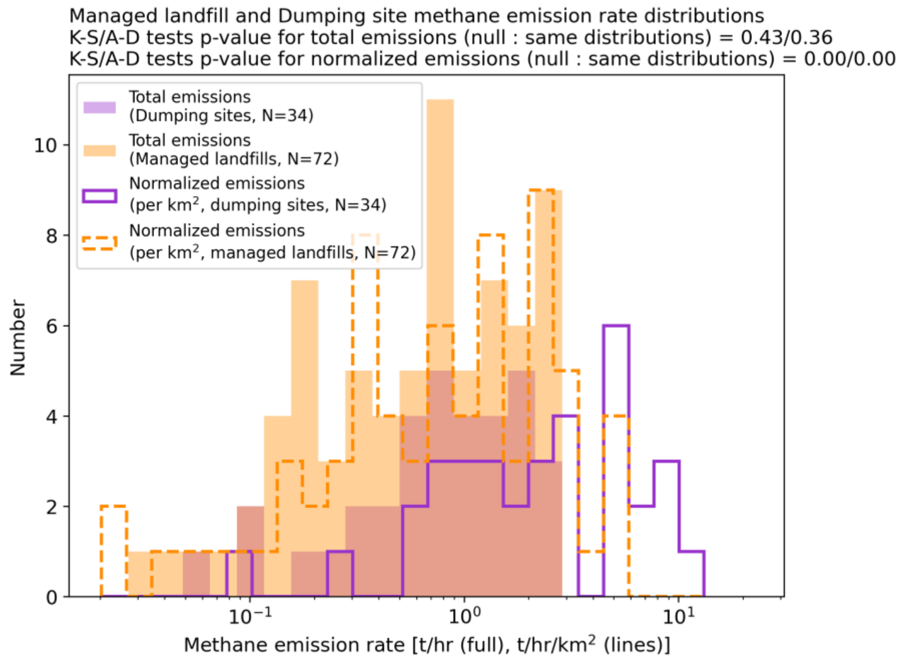

Supplementary Figure 12. Comparison of managed landfill and dumping site total and area-normalized emission distributions for sites emitting less than 2.5 t/hr

Distributions of total (full colors) and full-area-normalized (lines) methane emission rates for "managed landfills" (orange) and "dumping sites" (purple) following our classification, including only sites with emissions lower than 2.5 t/hr.

Supplementary Note 7: Bottom-up dataset filters

This supplement provides data source references and filtering for reported and calculated facility-scale methane emissions.

Supplementary Tables 5 – 8 report the data fields and values chosen to filter GHG emission reporting or calculation datasets.

US GHG Reporting program file names:

- 2022_data_summary_spreadsheets_0.zip, considering both files hereafter, and then computing either the mean, or using data from just one year depending on when GHGSat data have been observed:
  - ghgp_data_2021.xlsx
  - ghgp_data_2022.xlsx

US GHG Reporting program download link:

- (last accessed 2024-03-28) https://www.epa.gov/ghgreporting/data-sets

Data fields	Selected values
"Industry Type (subparts)"	'C,D,HH', 'C,DD,HH', 'C,HH', 'C,HH,TT', 'HH'

Supplementary Table 5. US GHGRP filters
Data fields and selected values used to select data from the US GHG Reporting.

The analysis is performed using the following data field that reports a yearly total in t of CO2e:

- 'Methane (CH4) emissions '

This total is converted to hourly rates assuming constant emissions, and using the AR4 CH4 GWP, as used by EPA (https://www.epa.gov/ghgreporting/ghgrp-reported-data, last accessed 2024-06-24).

Canadian GHG Reporting program file name:

- PDGES-GHGRP-GHGEmissionsGES-2004-Present.csv

Canadian GHG Reporting program download link:

- (last accessed 2024-06-06) https://open.canada.ca/data/en/dataset/a8ba14b7-7f23-462a-bdbb-83b0ef629823

**Data fields**	**Selected values**
"English Facility NAICS Code Description / Description du code SCIAN de l'installation en anglais"	'Waste Treatment and Disposal'<br>OR<br>'Waste Collection'<br>OR<br>'All Other Waste Management Services'
"Reference Year / Année de référence"	Considering both available values listed hereafter, and computing the mean, or using data from just one year depending on when GHGSat data have been observed:<br>• 2021<br>• 2022

Supplementary Table 6. Canadian GHGRP filters
Data fields and selected values used to select data from the Canadian GHG reporting program.

The analysis is performed using the following data field that reports a yearly total in metric tons of CH4:

- 'CH4 (tonnes)'

This total is converted to hourly rates assuming constant emissions.

European GHG Reporting program (E-PRTR) file name:

- F1_4_Detailed releases at facility level with E-PRTR Sector and Annex I Activity detail into Air.xlsx

European GHG Reporting program download link:

- (last accessed 2024-06-06) https://sdi.eea.europa.eu/data/63a14e09-d1f5-490d-80cf-6921e4e69551?path=%2FUser%20friendly%20Excel%20file

Data fields	Selected values
'targetRelease'	'AIR'
'pollutant'	'Methane (CH4)'
Considering both values hereafter and then computing the mean, or using data from just one year depending on when GHGSat data have been observed:<br>'2021'<br>'2022'	>0

Supplementary Table 7. European GHG reporting program filters
Data fields and selected values used to select data from the European GHG reporting program (E-PRTR).

The analysis is performed using the following data fields that report a yearly total in kg of CH_4:

- '2021' and/or '2022'

This total is converted to hourly rates assuming constant emissions.

Climate TRACE GHG emission calculation file name:

- solid-waste-disposal_emissions-sources.csv

Climate TRACE GHG emission calculation:

- https://climatetrace.org/data

Data fields	Selected values
gas	Ch4
'start_time'	Considering both available values listed hereafter, and computing the mean, or using data from just one year depending on when GHGSat data have been observed:<br>• '2021-01-01 00:00:00'<br>• '2022-01-01 00:00:00'

Supplementary Table 8. Climate TRACE filters
Data fields and selected values used to select data provided by Climate TRACE.

The analysis is performed using the following data field that reports a yearly total in metric tons of CH4:

- 'emissions_quantity'

This total is converted to hourly rates assuming constant emissions.

Supplementary Note 8: Supplementary results for GHGSat comparison to facility-scale reported and calculated emission rates

This Supplementary Note provides difference statistics for all GHGSat emission rates comparison to reported and modelled data (Supplementary Tables 9 and 10).

Reporting scope	Number of sites	GHGSat – reported difference	GHGSat totals	Reported totals
USA	22	0.42 ± 1.44 t/hr	32.4 ± 4.8 t/hr	23.1 t/hr (reported to GHGRP)<br>49.9 t/hr (using emission models)
Canada	8	-0.50 ± 0.92 t/hr	3.2 ± 0.6 t/hr	7.1 t/hr
EU	7	1.74 ± 1.22 t/hr	14.5 ± 1.5 t/hr	2.3 t/hr
All together	37	0.47 ± 1.49 t/hr	50.1 ± 5.1 t/hr	32.6 t/hr (with data reported to GHGRP)<br>59.3 t/hr (using reported emission models for US)

Supplementary Table 9. Methane emission rate statistics comparing GHGSat-based rates and data included in facility-scale reporting program databases

Waste disposal site types	Number of sites	GHGSat – Climate TRACE difference	GHGSat totals	Climate TRACE totals
Managed landfills	77	-0.64 ± 4.92 t/hr	170 ± 8 t/hr	219 t/hr
Dumping sites	32	-0.35 ± 2.93 t/hr	63 ± 6 t/hr	75 t/hr
All together	109	-0.56 ± 4.44 t/hr	233 ± 10 t/hr	294 t/hr

Supplementary Table 10. Methane emission rate statistics comparing GHGSat-based rates and data calculated by Climate TRACE

Supplementary Tables 9 and 10 show that the bias between GHGSat-based and reported emissions is generally much lower than the standard deviation of the differences, highlighting the poor correlations showcased in Figure 3 in the Main text. Considering the ~100% uncertainties reported bottom-up data can show (e.g. reported totals in the US depend on the chosen method), we do not find significantly different sums of emissions between bottom-up data and reported top-down across our sites. We interpret this result as an indication that our satellite-based results do not show significant systematic biases compared to bottom-up data across the dataset.

Supplementary Figure 13 reproduces Figure 3 only including sites that show at least 5 observations. The resulting correlations remain poor. Finally, Supplementary Figure 14 helps

to assess the robustness of our conclusions to the site-wise averaged emission rate uncertainties. For an ensemble of 1000 random draws following a normal distribution centered on GHGSat-based averaged site-wise emission rates and with a standard deviation matching the averaged emission uncertainties, we compute similar correlations as in Figure 3. Supplementary Figure 14 shows that correlations are consistently low across the random draws for both reported and Climate TRACE modelled bottom-up data, thus showing the robustness of our conclusions to emission rate uncertainties.

Given the sensitivity results provided in Supplementary Figures 13 and 14, the results shown in Figure 3 are robust to the number of available observations per site, and to the prescribed uncertainty ranges.

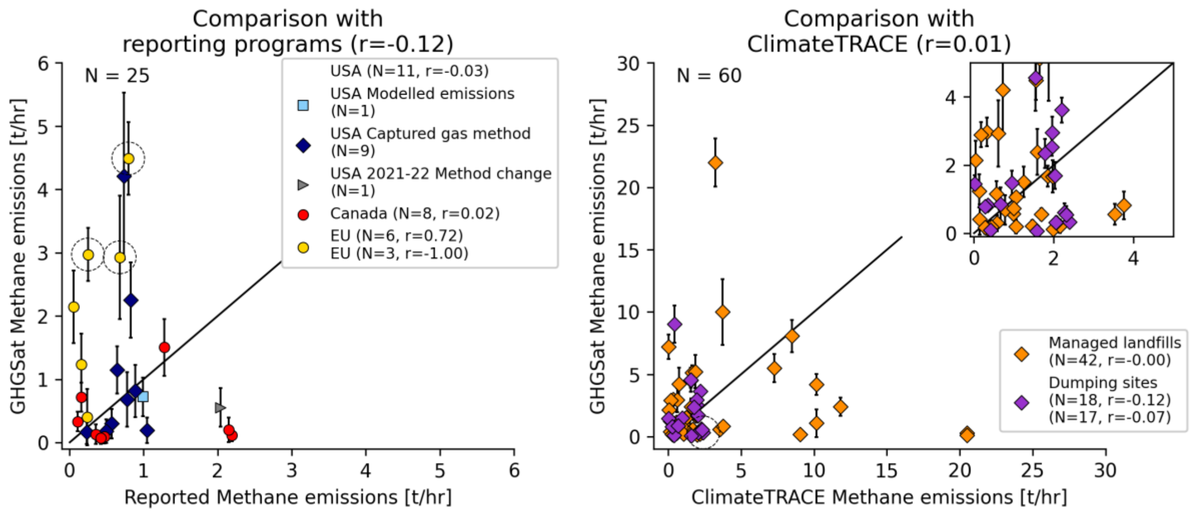

Supplementary Figure 13. Version of Figure 3 including only sites with at least 5 GHGSat observations

The conclusions remain unchanged.

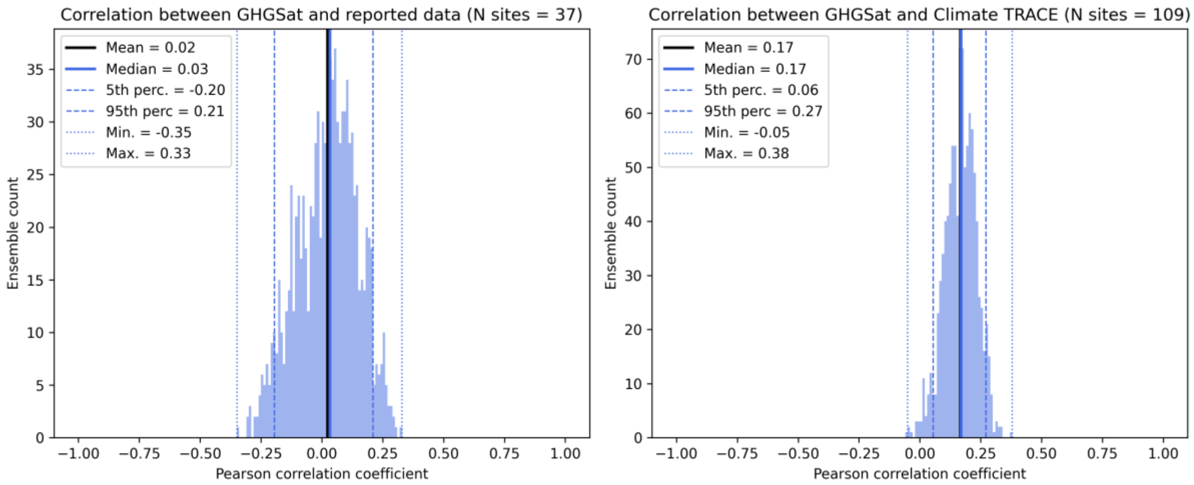

Supplementary Figure 14. Impact of site-wise emission uncertainty on correlations shown in Figure 3

Distribution of Pearson correlation coefficient between GHGSat-based emission rate and reported bottom-up values (left) and Climate TRACE modelled emissions (right) for an

ensemble of 1000 random draws for GHGSat-based emission rates, following a normal distribution based on site-wise averaged emission rates and their related uncertainties.
Finally, we also assess the sensitivity of our results to the emission rate range of the sampled sites. The 2.5 t/hr inflection point in the cumulative distribution of facility-level emissions in the Climate TRACE dataset (Extended Data Figure 7) indicates that sites emitting below this threshold account for the majority of total emissions. Consequently, we also discuss our comparison of GHGSat-based emission rates against reported or modelled data using only sites emitting below 2.5 t/hr. Supplementary Figure 15 reproduces Figure 3 for the 106/151 sites that emit below 2.5 t/hr. We note that our conclusions remain unchanged: correlations between top-down and bottom-up datasets remains low.

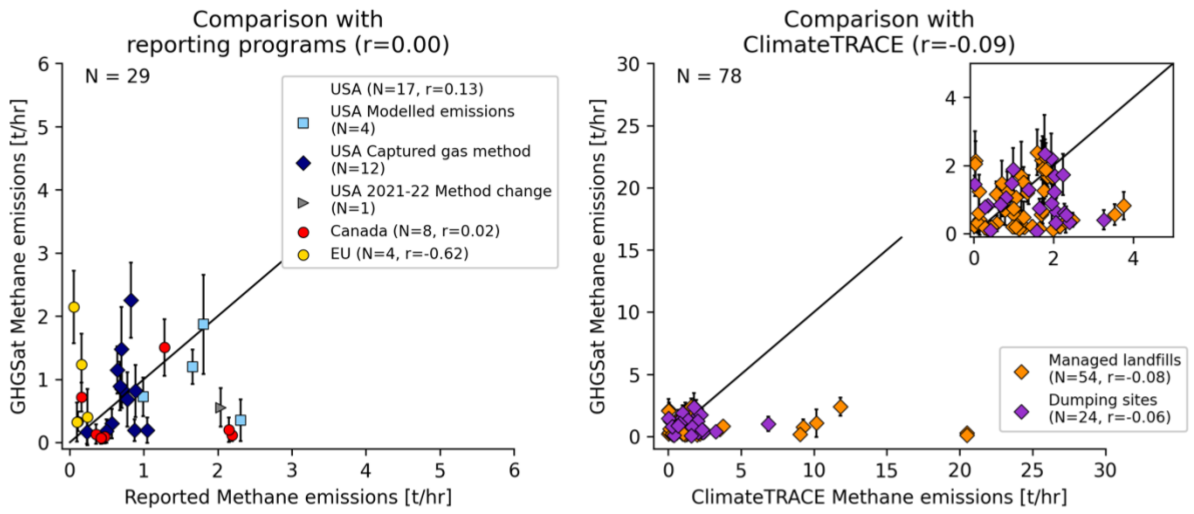

Supplementary Figure 15. Version of Figure 3 including only sites with averaged emission rates below 2.5 t/hr
The correlation between bottom-up and top-down estimates remain unchanged compared to the analysis of the full data set shown in Figure 3.

Supplementary Note 9: Analyzing the impact of reporting method choice for US facilities

The US Greenhouse Gas Reporting Program (GHGRP) requires that landfills provide annual methane emission estimates using two different methods: (1) based on gas capture efficiency; and (2) based on waste-decay modelling. Only one result is chosen by facility operators to be included in GHGRP data, but both are available on the 'Facility Level Information on GreenHouse gases Tool' (FLIGHT, https://ghgdata.epa.gov/ghgp/main.do, last access: 2025-08-07) platform, run by the US Environmental Protection Agency (EPA). Using identified facilities in the US, this supplement examines the reporting method impact on how GHGSat-based results compare to bottom-up estimates.

The Extended Data Figure 9 provides a comparison of GHGSat-based emission rates against official GHGRP facility-scale reported data (top left), and against data computed with the method based on gas capture efficiency (bottom left) and based on waste decay modelling (bottom right). Regardless of the reported data, all three comparisons in Extended Data Figure 9 exhibit low correlations between GHGSat-based emission estimates and reported data. The capture-based method leads to underestimating facility-scale emissions compared to GHGSat-based results, while waste-decay modelling appears to overestimate them. Both methods show a large scatter in how they compare to GHGSat.

Supplementary Note 10: Results of GHGSat plume sources comparison with Sentinel-2 detected surface activity

This supplement discusses the overview of GHGSat plume sources comparison with Sentinel-2 detected surface activity, and illustrates results for a few sites in addition to the Casablanca landfill shown in Figure 1.

Extended Data Figure 10 summarizes the results obtained for GHGSat plume source comparison to Sentinel-2 detected landfill surface activity over the 107 sites that passed all filtering criteria. We count 44/107 (41%) sites that show a statistically significant proximity (p-value<0.05) between GHGSat-detected plume sources and Sentinel-2 detected surface activity. The ratio of sites that show positive results increases when we restrict the analysis to sites where at least a given number of GHGSat-detected plume sources are available. For example, 18/21 (82%) sites that present at least 16 GHGSat-detected plume sources show statistically significant proximity (p-value<0.05). From these results, we conclude (1) that in many cases GHGSat-detected plumes are related to landfill surface activity; and (2) that increasing the number of observations over a given landfill helps to track emission sources within the facility, and more precisely pinpoint them if they are stationary.

Supplementary Figures 16 to 20 illustrate site-wise comparisons between GHGSat-detected plume sources and Sentinel-2 detected surface activity for different sites, as shown in Figure 1 for Casablanca landfill.

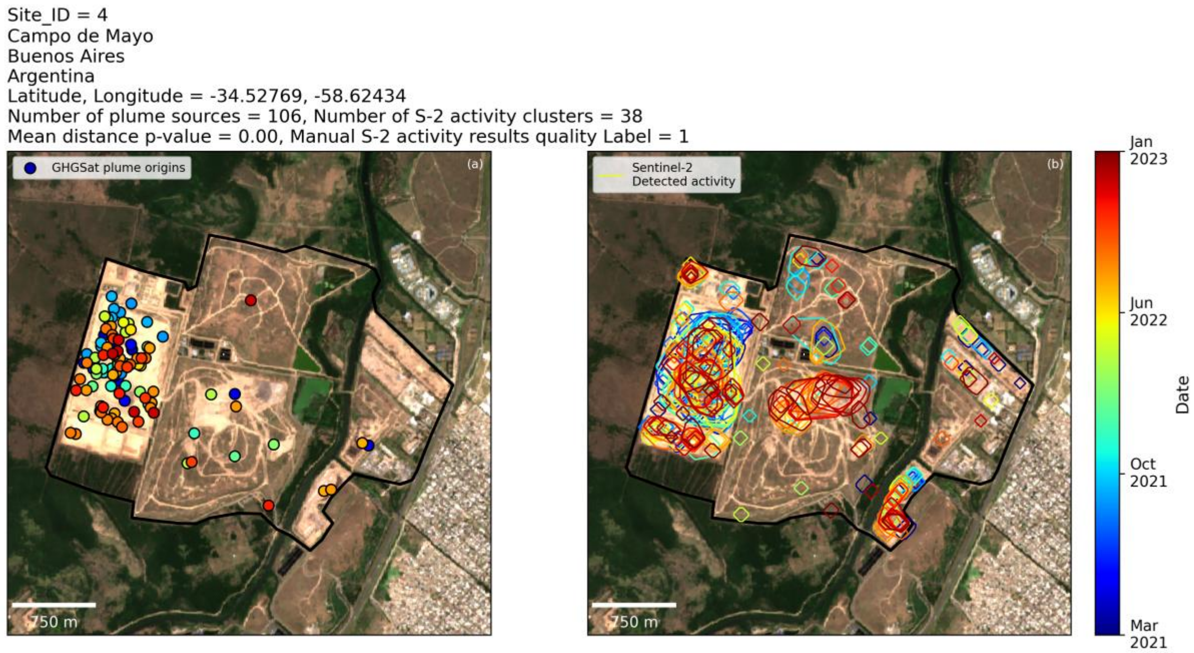

Supplementary Figure 16. Example of GHGSat-detected sources and Sentinel-2 detected activity in Buenos Aires (Argentina)

Comparison results for GHGSat-detected plume sources (left) and Sentinel-2 detected surface activity (right) at Norte III landfill, in Buenos Aires, Argentina. Background imagery relies on Sentinel-2 data (2022) sampled using the Google Earth Engine[36].

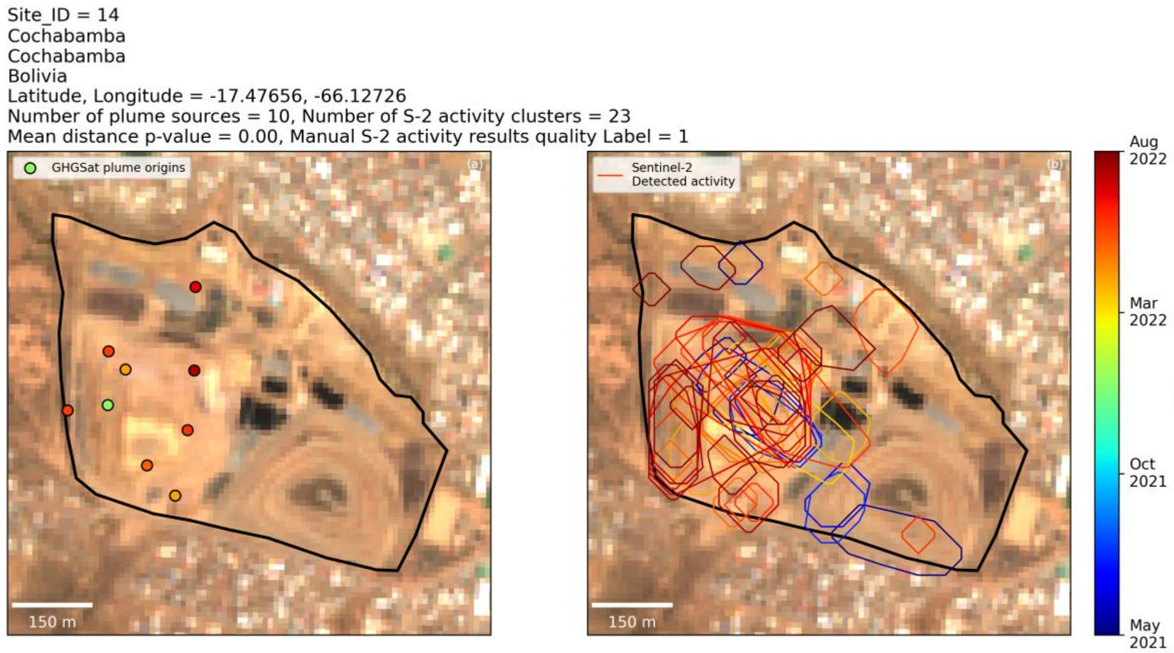

Supplementary Figure 17. Example of GHGSat-detected sources and Sentinel-2 detected activity in Cochabamba (Bolivia)

Comparison results for GHGSat-detected plume sources (left) and Sentinel-2 detected surface activity (right) at Cochabamba landfill, in Bolivia. Background imagery relies on Sentinel-2 data (2022) sampled using the Google Earth Engine[36].

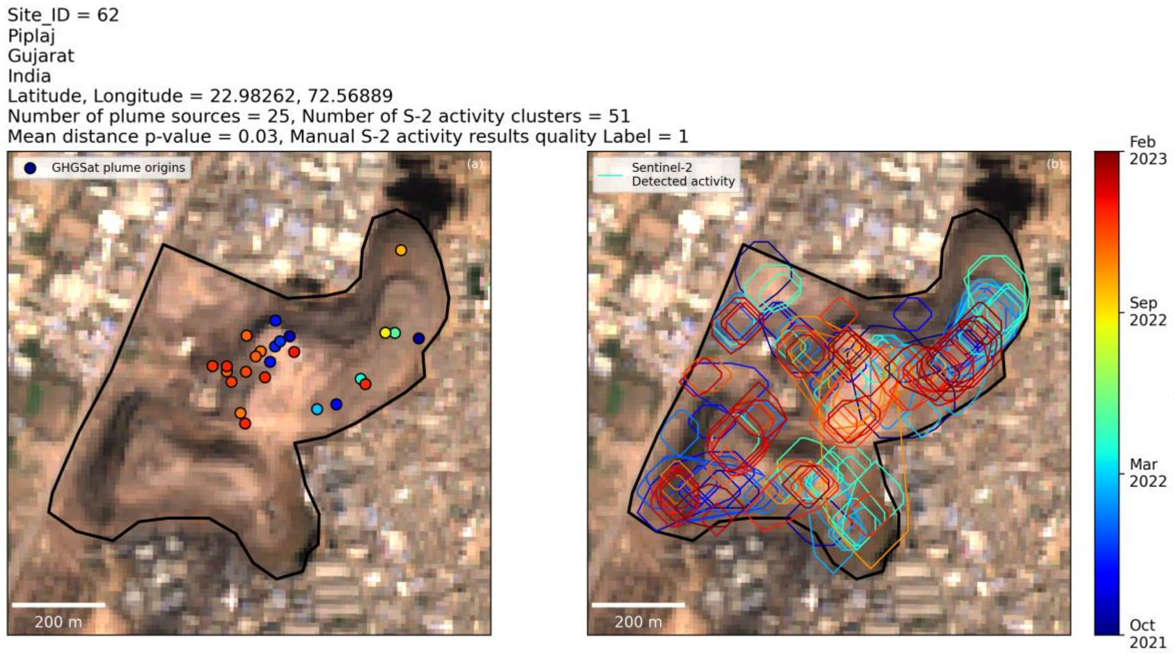

Supplementary Figure 18. Example of GHGSat-detected sources and Sentinel-2 detected activity near Ahmedabad (India)

Comparison results for GHGSat-detected plume sources (left) and Sentinel-2 detected surface activity (right) at a landfill near Ahmedabad, in India. Background imagery relies on Sentinel-2 data (2022) sampled using the Google Earth Engine[36].

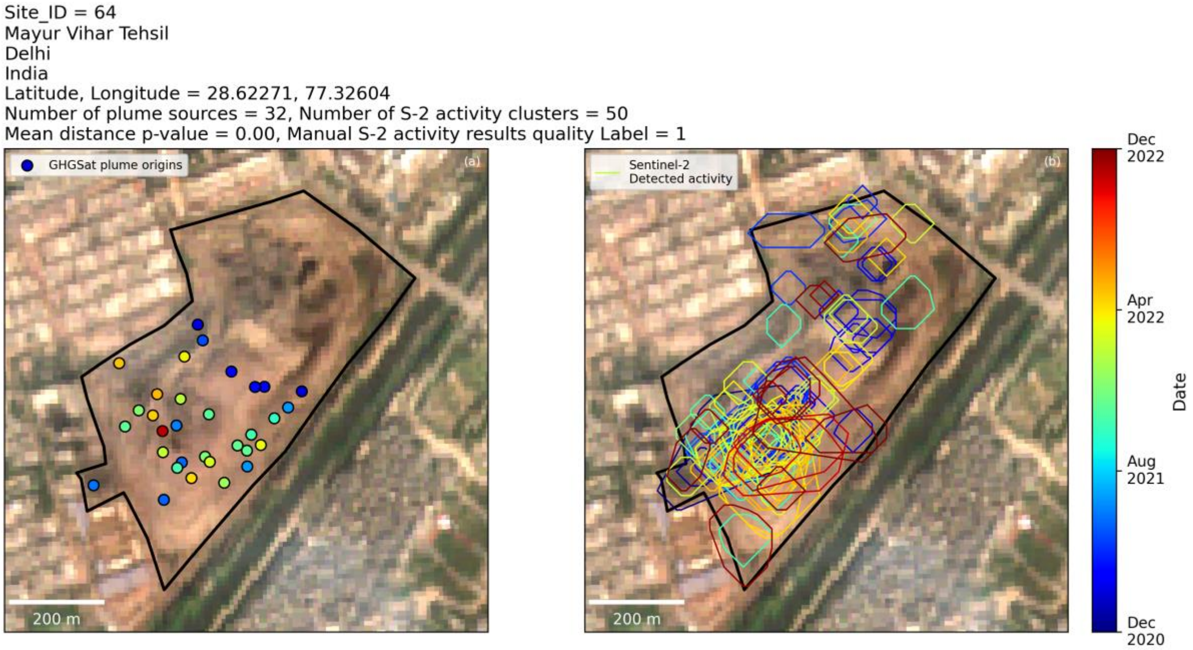

Supplementary Figure 19. Example of GHGSat-detected sources and Sentinel-2 detected activity in Dehli (India)

Comparison results for GHGSat-detected plume sources (left) and Sentinel-2 detected surface activity (right) at Ghazipur landfill, in Delhi, India. Background imagery relies on Sentinel-2 data (2022) sampled using the Google Earth Engine[36].

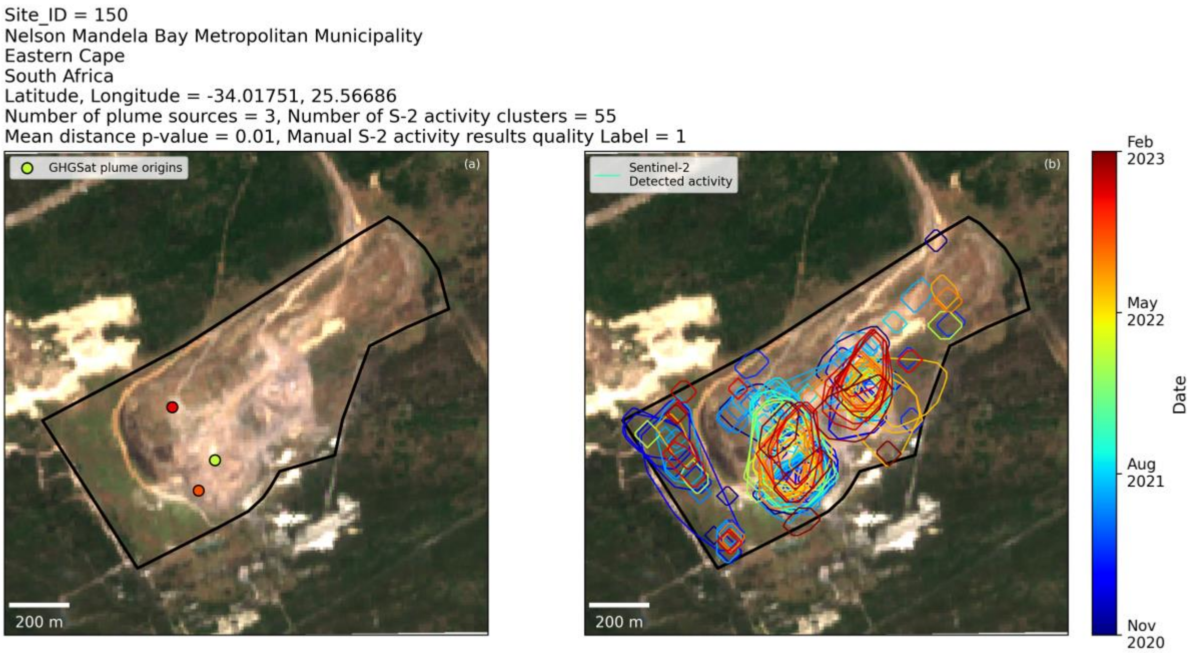

Supplementary Figure 20. Example of GHGSat-detected sources and Sentinel-2 detected activity in Nelson Mandela Bay Municipality (South Africa)

Comparison results for GHGSat-detected plume sources (left) and Sentinel-2 detected surface activity (right) at Nelson Mandela Bay Municipality landfill, in South Africa. Background imagery relies on Sentinel-2 data (2022) sampled using the Google Earth Engine[36].

Supplementary Note 11: example of detected plumes arising from other facilities than landfills

Supplementary Figures 21 and 22 show methane emission plumes arising from a wastewater treatment plant in Shanghai and from a biogas plant in Madrid, respectively.

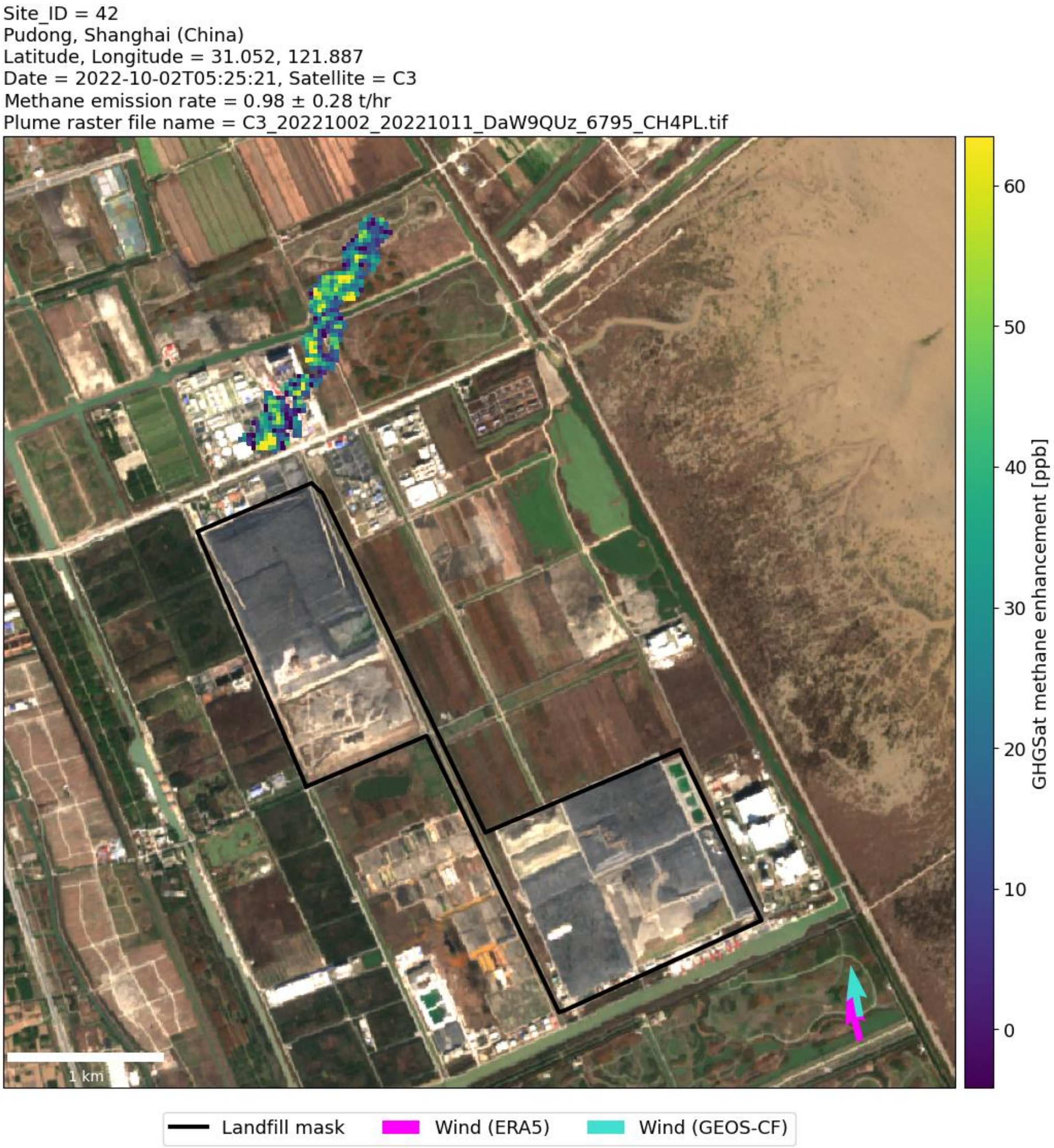

Supplementary Figure 21. Example of methane emission plume from a wastewater treatment plant

Methane emission plume arising from a wastewater treatment plant located close to the targeted landfill near Shanghai, and observed by GHGSat's C3 satellite on 2022 Oct 2nd. Background imagery relies on non-concurrent Sentinel-2 data (2022) sampled using the Google Earth Engine[36].

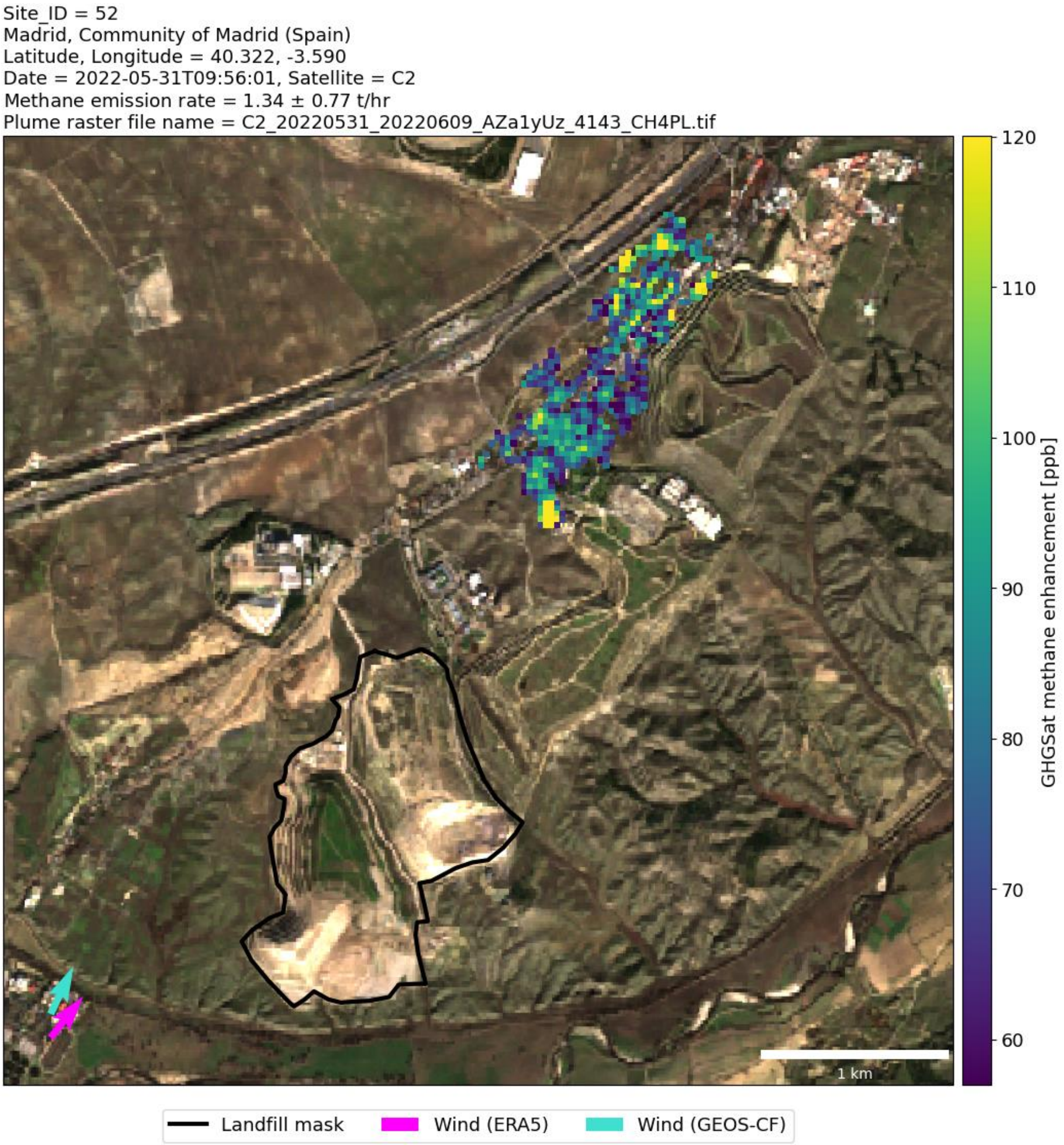

Supplementary Figure 22. Example of methane emission plume from a biogas processing plant

Methane emission plume arising from a biogas plant located close to a targeted landfill near Madrid, and observed by GHGSat's C2 satellite on 2022 May 31st. Background imagery relies on non-concurrent Sentinel-2 data (2022) sampled using the Google Earth Engine[36].
