# Supplementary material for: Global satellite survey reveals uncertainty in landfill methane emissions
Source: Nature. 2025 Nov 5;647(8089):397–402. doi: 10.1038/s41586-025-09683-8 (PMC12611785; doi:10.1038/s41586-025-09683-8)
Supplement: Supplementary file 2 — Reporting Summary [file 41586_2025_9683_MOESM2_ESM.pdf]

nature portfolio

Corresponding author(s): Matthieu Dogniaux

Last updated by author(s): 3 sept, 2025

Reporting Summary

Nature Portfolio wishes to improve the reproducibility of the work that we publish. This form provides structure for consistency and transparency in reporting. For further information on Nature Portfolio policies, see our Editorial Policies and the Editorial Policy Checklist.

Statistics

For all statistical analyses, confirm that the following items are present in the figure legend, table legend, main text, or Methods section.

n/a	Confirmed	
☐	☒	The exact sample size (*n*) for each experimental group/condition, given as a discrete number and unit of measurement
☒	☐	A statement on whether measurements were taken from distinct samples or whether the same sample was measured repeatedly
☐	☒	The statistical test(s) used AND whether they are one- or two-sided<br>*Only common tests should be described solely by name; describe more complex techniques in the Methods section.*
☒	☐	A description of all covariates tested
☒	☐	A description of any assumptions or corrections, such as tests of normality and adjustment for multiple comparisons
☐	☒	A full description of the statistical parameters including central tendency (e.g. means) or other basic estimates (e.g. regression coefficient) AND variation (e.g. standard deviation) or associated estimates of uncertainty (e.g. confidence intervals)
☐	☒	For null hypothesis testing, the test statistic (e.g. *F*, *t*, *r*) with confidence intervals, effect sizes, degrees of freedom and *P* value noted<br>*Give P values as exact values whenever suitable.*
☒	☐	For Bayesian analysis, information on the choice of priors and Markov chain Monte Carlo settings
☒	☐	For hierarchical and complex designs, identification of the appropriate level for tests and full reporting of outcomes
☐	☒	Estimates of effect sizes (e.g. Cohen's *d*, Pearson's *r*), indicating how they were calculated

Our web collection on statistics for biologists contains articles on many of the points above.

Software and code

Policy information about availability of computer code

Data collection	We use the well-established L2 (concentration) fields from ESA (Lorente et al., 2021) and GHGSat (Jervis et al., 2021) as starting point for the study<br><br>Lorente, A. et al.: Methane retrieved from TROPOMI: improvement of the data product and validation of the first 2 years of measurements, Atmos. Meas. Tech., 14, 665–684, https://doi.org/10.5194/amt-14-665-2021, 2021.<br><br>Jervis, D. et al.: The GHGSat-D imaging spectrometer, Atmos. Meas. Tech., 14, 2127–2140, https://doi.org/10.5194/amt-14-2127-2021, 2021
Data analysis	Custom code was used, available on Code Ocean: https://codeocean.com/capsule/2078268/tree

For manuscripts utilizing custom algorithms or software that are central to the research but not yet described in published literature, software must be made available to editors and reviewers. We strongly encourage code deposition in a community repository (e.g. GitHub). See the Nature Portfolio guidelines for submitting code & software for further information.

Data

Policy information about availability of data

All manuscripts must include a data availability statement. This statement should provide the following information, where applicable:

- Accession codes, unique identifiers, or web links for publicly available datasets
- A description of any restrictions on data availability
- For clinical datasets or third party data, please ensure that the statement adheres to our policy

The Sentinel-5P TROPOMI data and Sentinel-2 data are available at the Copernicus Data Hub (https://dataspace.copernicus.eu). GEOS FP wind data can be downloaded from https://gmao.gsfc.nasa.gov/GMAO_products/. ERA5 and GEOS-CF meteorological data were sampled using Google Earth Engine. The GHGSat-detected methane plumes are available via Zenodo at: https://doi.org/10.5281/zenodo.16641834. Tables summarizing the results at site level for GHGSat, and at urban area level for TROPOMI are already included in the Supplementary Notes.

Research involving human participants, their data, or biological material

Policy information about studies with human participants or human data. See also policy information about sex, gender (identity/presentation), and sexual orientation and race, ethnicity and racism.

Reporting on sex and gender	Does not apply
Reporting on race, ethnicity, or other socially relevant groupings	Does not apply
Population characteristics	Does not apply
Recruitment	Does not apply
Ethics oversight	Does not apply

Note that full information on the approval of the study protocol must also be provided in the manuscript.

Field-specific reporting

Please select the one below that is the best fit for your research. If you are not sure, read the appropriate sections before making your selection.

☐ Life sciences ☐ Behavioural & social sciences ☒ Ecological, evolutionary & environmental sciences

For a reference copy of the document with all sections, see nature.com/documents/nr-reporting-summary-flat.pdf

Ecological, evolutionary & environmental sciences study design

All studies must disclose on these points even when the disclosure is negative.

Study description	Satellite survey of landfill methane emissions and associated urban methane emissions
Research sample	151 waste disposal sites located across all inhabited continents for which successful GHGSat observations of methane plumes were available. Associated TROPOMI analysis of data over those areas
Sampling strategy	TROPOMI uses a continuous global sampling strategy while GHGSat performs targeted observations
Data collection	Satellite observation
Timing and spatial scale	Global scale observation set over 2021 and 2022
Data exclusions	For TROPOMI we only use cloud-free observations to detect urban plumes using an automated approach as described in Schuit et al. (2023). For GHGSat, only clear-sky observations suitable for emission quantification were used<br><br>Schuit, B. J. et al.: Automated detection and monitoring of methane super-emitters using satellite data, Atmos. Chem. Phys., 23, 9071–9098, https://doi.org/10.5194/acp-23-9071-2023, 2023
Reproducibility	The Code Ocean repository allows to reproduce the analysis
Randomization	Does not apply
Blinding	Does not apply

Did the study involve field work? ☐ Yes ☒ No

Reporting for specific materials, systems and methods

We require information from authors about some types of materials, experimental systems and methods used in many studies. Here, indicate whether each material, system or method listed is relevant to your study. If you are not sure if a list item applies to your research, read the appropriate section before selecting a response.

Materials & experimental systems

n/a	Involved in the study
☒	☐ Antibodies
☒	☐ Eukaryotic cell lines
☒	☐ Palaeontology and archaeology
☒	☐ Animals and other organisms
☒	☐ Clinical data
☒	☐ Dual use research of concern
☒	☐ Plants

Methods

n/a	Involved in the study
☒	☐ ChIP-seq
☒	☐ Flow cytometry
☒	☐ MRI-based neuroimaging

Plants

Seed stocks	*Report on the source of all seed stocks or other plant material used. If applicable, state the seed stock centre and catalogue number. If plant specimens were collected from the field, describe the collection location, date and sampling procedures.*
Novel plant genotypes	*Describe the methods by which all novel plant genotypes were produced. This includes those generated by transgenic approaches, gene editing, chemical/radiation-based mutagenesis and hybridization. For transgenic lines, describe the transformation method, the number of independent lines analyzed and the generation upon which experiments were performed. For gene-edited lines, describe the editor used, the endogenous sequence targeted for editing, the targeting guide RNA sequence (if applicable) and how the editor was applied.*
Authentication	*Describe any authentication procedures for each seed stock used or novel genotype generated. Describe any experiments used to assess the effect of a mutation and, where applicable, how potential secondary effects (e.g. second site T-DNA insertions, mosiacism, off-target gene editing) were examined.*
